# PI3K-C2γ is a Rab5 effector selectively controlling endosomal Akt2 activation downstream of insulin signalling

**DOI:** 10.1038/ncomms8400

**Published:** 2015-06-23

**Authors:** Laura Braccini, Elisa Ciraolo, Carlo C. Campa, Alessia Perino, Dario L. Longo, Gianpaolo Tibolla, Marco Pregnolato, Yanyan Cao, Beatrice Tassone, Federico Damilano, Muriel Laffargue, Enzo Calautti, Marco Falasca, Giuseppe D. Norata, Jonathan M. Backer, Emilio Hirsch

**Affiliations:** 1 Molecular Biotechnology Center, Department of Molecular Biotechnology and Health Sciences, University of Torino, Torino 10126, Italy; 2 Department of Pharmacological and Biomolecular Sciences, University of Milan, Milan 20133, Italy; 3 Department of Molecular Pharmacology, Albert Einstein College of Medicine, Bronx, New York, New York 10461, USA; 4 INSERM UMR 1048, I2MC, Bât. L3, 1 av Jean-Poulhès, BP 84225, Toulouse 4 31432, France; 5 Metabolic Signalling Group, School of Biomedical Sciences, CHIRI Biosciences, Curtin University, Perth, Western Australia 6102, Australia

## Abstract

In the liver, insulin-mediated activation of the phosphatidylinositol 3-kinase (PI3K)/Akt pathway is at the core of metabolic control. Multiple PI3K and Akt isoenzymes are found in hepatocytes and whether isoform-selective interplays exist is currently unclear. Here we report that insulin signalling triggers the association of the liver-specific class II PI3K isoform γ (PI3K-C2γ) with Rab5-GTP, and its recruitment to Rab5-positive early endosomes. In these vesicles, PI3K-C2γ produces a phosphatidylinositol-3,4-bisphosphate pool specifically required for delayed and sustained endosomal Akt2 stimulation. Accordingly, loss of PI3K-C2γ does not affect insulin-dependent Akt1 activation as well as S6K and FoxO1-3 phosphorylation, but selectively reduces Akt2 activation, which specifically inhibits glycogen synthase activity. As a consequence, PI3K-C2γ-deficient mice display severely reduced liver accumulation of glycogen and develop hyperlipidemia, adiposity as well as insulin resistance with age or after consumption of a high-fat diet. Our data indicate PI3K-C2γ supports an isoenzyme-specific forking of insulin-mediated signal transduction to an endosomal pool of Akt2, required for glucose homeostasis.

## Abstract (web summary)

The kinase PI3K is crucial for insulin signalling in the liver but the roles of individual PI3K isoforms are largely unclear. Using mice that lack class II PI3K isoform γ (PI3K-C2γ), the authors here show that PI3K-C2γ selectively activates endosomal Akt2 by regulating the localized production of PIP2.

## Graphical abstract

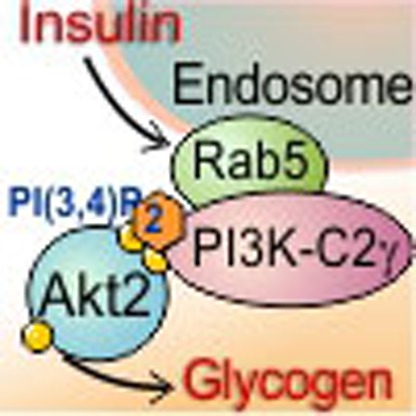

Downstream insulin receptor activation, phosphatidylinositol 3-kinases (PI3Ks) cause the acute accumulation on the plasma membrane of two lipid second messengers: phosphatidylinositol-3,4,5-trisphosphate (PtdIns(3,4,5)*P*_3_) and phosphatidylinositol-3,4-bisphosphate (PtdIns(3,4)*P*_2_). These two molecules act as docking sites for signal amplifiers like the serine/threonine kinase Akt that, upon its association to either PtdIns(3,4,5)*P*_3_ or PtdIns(3,4)*P*_2_, is phosphorylated on two key residues (Thr308/309 and Ser473/474 in Akt1/Akt2, respectively) and activated^1^. Akt, in turn, promotes multiple insulin-dependent cellular responses by phosphorylating a plethora of target proteins modulating glucose and triglyceride hepatic metabolism^2^. Although this acutely occurs at the plasma membrane and mainly involves class I PI3Kα^3^, a delayed but sustained response can propagate from internal membranes as well. After agonist binding, the activated insulin receptor is internalized into endosomes from which it is either recycled to the plasma membrane or degraded through lysosome activity. Although internalization is a key event for signal termination, endocytosis also contributes to prolong Akt phosphorylation beyond PI3K activation at the plasma membrane^4^. In line with this view, the depletion of Rab5, a master regulator of endosome biogenesis, significantly reduces phosphorylation of Akt upon insulin stimulation^5^. Downstream of Rab5, Akt phosphorylation/activation outside the plasma membrane further requires the adaptor protein containing PH domain, PTB domain and Leucine zipper motif (APPL1) that acts as a Rab5 effector promoting Akt recruitment and activation on early endosomes (EEs)^6^. APPL1 is thought to release Akt from the endogenous inhibitor protein tribble 3 (TRB3) and to target it to endosomal membranes^6^. Nonetheless, the precise nature of the lipid and the PI3K involved in endocytosis-dependent Akt activation is still unclear.

Although class I PI3Kβ is involved in insulin signalling^7^,^8^ and acts as a Rab5 effector^9^,^10^,^11^, its function appears related to the production of a PtdIns(3,4,5)*P*_3_ pool that is rapidly converted into PtdIns3P, which in turn induces APPL1 release from endosomes, thus terminating the signalling cascade^10^,^12^. Given the preferential localization of PtdIns(3,4,5)*P*_3_ at the plasma membrane, the PI3K product promoting Akt activation in endosomes appears to be PtdIns(3,4)*P*_2_, which is known to be abundant in early endocytic membranes^13^. Although PtdIns(3,4)*P*_2_ can be produced from PtdIns(3,4,5)*P*_3_ by the action of the enzyme 5-phosphatase, Src homology 2 domain containing inositol phosphatase 2 (SHIP2), other potential sources of this lipid are the three class II PI3Ks (PI3K-C2α, β and γ), which are known to produce *in vitro* both PtdIns(3,4)*P*_2_ and PtdIns3P. In line with this view, PI3K-C2α has been found to promote endocytosis by producing a pool of PtdIns(3,4)*P*_2_ on clathrin-coated pits that is crucial to recruit Sorting nexin 9 and dynamin, two key elements supporting the maturation of pits into endocytic vesicles^14^. Although this indicates that class II PI3Ks can produce PtdIns(3,4)*P*_2_ *in vivo*, this ubiquitous class II PI3K is not known to participate in Akt activation but rather in other pathways of insulin signalling such as GLUT4 translocation in muscle cells^15^,^16^, or unrelated processes such as exocytosis of insulin granules in pancreatic cells^17^ as well as organization of the recycling compartment^18^. Similar to PI3K-C2α, PI3K-C2β has as yet not been reported to play major role in Akt activation. Although PI3K-C2β can be activated downstream of several tyrosine kinase receptors, such as the EGFR, ErbB2 and PDGFR^19^, this isoform is only weakly involved in insulin signalling^20^, and consistently, PI3K-C2β-deficient mice are not prone to insulin resistance^21^. On the other hand, little is known about PI3K-C2γ that, differently from the ubiquitous PI3K-C2α and PI3K-C2β, is specifically enriched in liver parenchyma cells^22^,^23^. Like other class II PI3Ks, PI3K-C2γ catalyses the synthesis of PtdIns3P and PtdIns(3,4)*P*_2_ *in vitro*^24^ but the specific lipid produced *in vivo* is still unclear. However, human genetics studies link the PI3K-C2γ gene with insulin signalling, showing an association between a polymorphism in the PI3K-C2γ-encoding gene (*PIK3C2G*) *Pik3c2g* and increased incidence of type 2 diabetes mellitus in a set of Japanese patients^25^.

Here we show that PI3K-C2γ is dispensable for insulin-dependent acute Akt phosphorylation but that this lipid kinase plays a major role to selectively support long-term Akt2 activation in intracellular vesicles. PI3K-C2γ is recruited by Rab5-GTP to EEs where it promotes PtdIns(3,4)*P*_2_ accumulation and the activation of Akt2. This impacts on the signalling to specific subcellular targets such as glycogen synthase (GS but not to other Akt substrates like S6K or FoxO1-3 transcription factors). In the absence of this specific signal transduction branch, the mouse liver fails to accumulate glycogen and promotes a shunt towards triglycerides production, thus triggering age- or diet-related insulin resistance and adiposity.

## Results

### PI3K-C2γ is preferentially expressed in the liver

**Figure 1:**
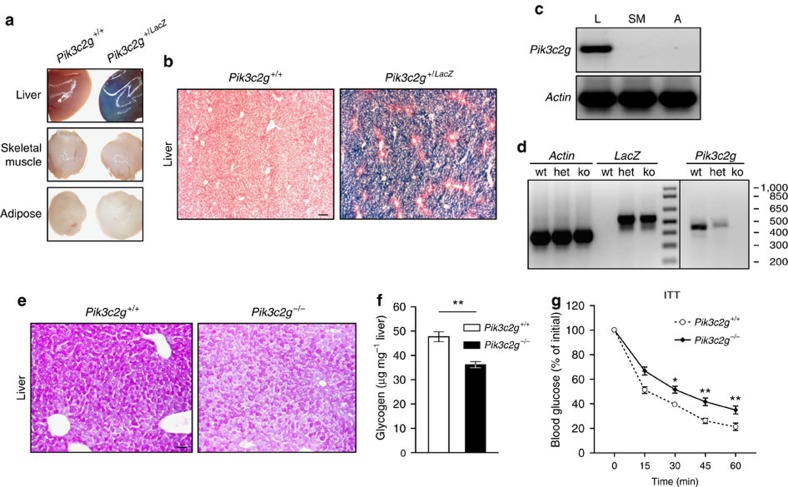
PI3K-C2γ is enriched in the liver and its absence leads to reduced hepatic glycogen. (**a**) Whole-mount LacZ staining of organs derived from heterozygous mutant (*Pik3c2g*^*+/LacZ*^) mice and controls (*Pik3c2g*^*+/+*^) imaged *in toto*. (**b**) Representative LacZ staining of liver sections from 2-month-old *Pik3c2g*^*+/+*^ and heterozygous *Pik3c2g*^*+/−*^ mice. Scale bar, 100 μm. (**c**) RT–PCR analysis of *Pik3c2g* expression in prominent insulin-sensitive organs (A, adipose tissue; L, liver; SM, skeletal muscle). Actin is used as loading control. (**d**) RT–PCR analysis of *Pik3c2g, Actin and LacZ* mRNA from liver extract of wild-type (wt), heterozygous (het) and knockout mice (ko). Molecular weight markers are indicated on the right. (**e**) Representative histological liver sections stained with periodate-Schiff, evidencing carbohydrate deposits in hepatocytes. Scale bar, 40 μm. (**f**) Hepatic glycogen content, determined by acid hydrolysis in livers obtained from 2-month-old re-fed mice (overnight fast followed by 4 h refeeding; *n*=6 and *n*=4, respectively). (**g**) Insulin tolerance test (ITT) in 2-month-old *Pik3c2g*^*+/+*^ and *Pik3c2g*^*−/−*^ mice (*n*=6 and *n*=11, respectively). Glucose levels are reported as a percentage of the starting value at the time of insulin injection. Results represent mean±s.e.m. *P<0.05, **P<0.01 mutant versus the respective wild-type controls. *P* values were determined using Student’s *t*-test (**f**) and two-way analysis of variance followed by Bonferroni *post-hoc* test (**g**).

A mouse carrying the *lacZ* reporter gene in frame with the first ATG codon of the *Pik3c2g* gene was generated by standard gene targeting technology (*Pik3c2g*^*+/lacZ*^, Supplementary Fig. 1a,b). In agreement with previous gene expression studies^22^, *Pik3c2g*^*+/lacZ*^ mice displayed β-galactosidase expression restricted to the liver (Fig. 1a). Traces also appeared in the pancreas (Supplementary Fig. 1c). No expression was detected in classical insulin-sensitive tissues such as skeletal muscle and fat deposits (Fig. 1a) or in several other organs, including the brain (Supplementary Fig. 1c). Histological analysis of liver sections revealed that β-galactosidase expression was localized to hepatic parenchyma (Fig. 1b). In the pancreas, prolonged LacZ staining revealed expression in exocrine acinar cells but not in insulin producing islets of Langerhans (Supplementary Fig. 1d). Reverse transcription (RT)–PCR analysis confirmed hepatic expression but no traces of the *Pik3c2g* transcript were found in the other major insulin-sensitive tissues like skeletal muscle and adipose tissue (Fig. 1c).

To gain deeper insight into the function of PI3K-C2γ, heterozygous mice (*Pik3c2g*^*+/lacZ*^) were mated to obtain homozygous offspring (*Pik3c2g*^*lacZ/lacZ*^). *Pik3c2g*^*lacZ/lacZ*^ mice were born at the expected Mendelian ratio and were confirmed to lack *Pik3c2g* mRNA expression (Fig. 1d). *Pik3c2g*^*lacZ/lacZ*^ (for simplicity, further referred to as *Pik3c2g*^*−/−*^) mice appeared indistinguishable from their wild-type littermates at birth, did not show any growth retardation and normally reached adulthood. Furthermore, microscopic architecture of the liver parenchyma appeared normal and assessment of a marker of hepatic function, such as albumin, did not show any difference between 2-month-old mutant and control mice (*Pik3c2g*^*−/−*^ 3.01±0.07 g dl^−1^ versus *Pik3c2g*^*+/+*^ 3.13±0.10 g dl^−1^).

### Defective insulin response in *Pik3c2g* ^*−/−*^ mice

Histopathological assessment of liver sections revealed reduced positivity to periodic acid–Schiff staining in mutant samples (Fig. 1e), suggesting a decrease in glycogen deposits in *Pik3c2g*^*−/−*^ livers. In further agreement, a significant 25% reduction in glycogen was observed by biochemical determination (Fig. 1f). This was accompanied by ∼20% reduction in liver weight (1.12±0.067 g in *Pik3c2g*^*−/−*^ versus 1.419±0.063 g in *Pik3c2g*^*+/+*^ mice; Supplementary Fig. 1e). Given that reduction of glycogen deposits is often associated to insulin resistance^26^,^27^,^28^, mutant mice were tested for alterations in the insulin response. In agreement with a role of *Pik3c2g* in insulin-mediated control of glucose metabolism, the insulin tolerance test (ITT) in 2-month-old *Pik3c2g*^*−/−*^ mice showed significantly lower insulin sensitivity than in wild-type controls (Fig. 1g).

In addition to abnormal ITT and reduced glycogen deposits, mutant mice showed increased fat storage, as epididymal fat pads from mutant mice were ∼30% heavier than those from wild-type controls (0.24±0.018 g in P*ik3c2g*^*−/−*^ versus 0.1620±0.011 g in *Pik3c2g*^*+/+*^ mice; Supplementary Fig. 1e). This was specific to adipose tissue as other insulin responsive organs, such as skeletal muscles, did not show weight changes (Supplementary Fig. 1e). Furthermore, sections of epididymal fat pads showed a 30% enlargement of the adipocyte area in mutant cells (Supplementary Fig. 1f). This difference was unrelated to changes in food intake (3.4±0.4 g per day in *Pik3c2g*^*−/−*^ versus 3.6±0.1 g per day in *Pik3c2g*^*+/+*^ mice, *P*=not significant (Student’s *t*-test)) or physical activity (69,501±7,077 in *Pik3c2g*^*−/−*^ versus 73,930±3,054 in *Pik3c2g*^*+/+*^ mice, reported as movements over a 24-h period in an activity cage). In line with altered lipid metabolism, although plasma cholesterol levels were unchanged, triglyceride levels were significantly higher in *Pik3c2g*^*−/−*^ mice than in controls (Supplementary Fig. 1g). These observations thus indicate that PI3K-C2γ plays a specific role in hepatic glycogen accumulation. In its absence, a modification in lipid production and storage occurs as a likely compensatory effect, in response to impaired insulin responses in the liver.

### PI3K-C2γ sustains hepatic Akt2 phosphorylation

**Figure 2:**
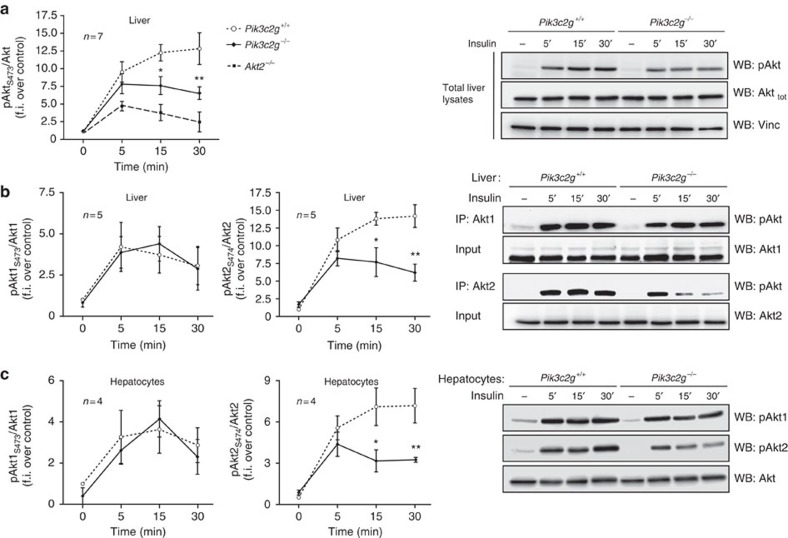
Loss of PI3K-C2γ reduces prolonged and sustained hepatic Akt2 phosphorylation. (**a**) Western blot analysis (right panel) of Akt phosphorylation in liver extracts from 2-month-old *Pik3c2g*^*+/+*^ and *Pik3c2g*^*−/−*^ mice injected with vehicle or insulin. The immunoblot of *Pik3c2g*^*+/+*^ and *Pik3c2g*^*−/−*^ samples is representative of seven independent experiments. Quantitative densitometry of western blots (WBs) is represented (*n*=7, left panels) and include the analysis of *Akt2*^*−/−*^ liver samples shown in Supplementary Fig. 3a. F.i: fold induction over unstimulated *Pik3c2g*^*+/+*^ control of pAkt values normalized to total Akt. (**b**) Analysis of Akt1 and Akt2 phosphorylation. Protein extracts from liver of insulin-injected mice were immunoprecipitated (IP) with anti-Akt1 or anti-Akt2 antibody and immunoblotted (WB) with anti-pSer473-Akt antibody. Immunoblots are representative of five independent experiments (right panels). Quantitative densitometry of WBs is represented (*n*=5, left panels). (**c**) Analysis of Akt1 and Akt2 phosphorylation in primary hepatocytes. Protein extracts of stimulated cells were immunoblotted with distinct antibodies selective for the phosphorylated Ser473 or Ser474 Akt1 or Akt2 isoform, respectively. Immunoblots are representative of four independent cell preparations (right panel). Quantitative densitometry of WBs is represented (*n*=4, left panels). Results represent mean±s.e.m of the number of replicates (*n*) reported in the figure. *P<0.05, **P<0.01 mutant versus the respective wild-type controls. *P* values were determined using two-way analysis of variance followed by Bonferroni *post-hoc* test.

Given the role of the PI3K/Akt axis in insulin signalling, the defective insulin-dependent responses detected in 2-month-old mutant mice suggest a role for PI3K-C2γ in Akt activation. To test if PI3K-C2γ was involved in the control of Akt activation, Akt phosphorylation in response to insulin was studied in liver extracts derived from 2-month-old mutant and control mice stimulated *in vivo* with insulin and collected at different time points after agonist administration. At 5 min after stimulation, Akt phosphorylation at the hydrophobic motif (Ser473 or 474 in Akt1 and 2, respectively) showed a similar increase in both *Pik3c2g*^*−/−*^ and *Pik3c2g*^*+/+*^ samples (Fig. 2a). On the contrary, Akt phosphorylation rapidly declined in *Pik3c2g*^*−/−*^ livers at 15 and 30 min after stimulation, whereas it kept increasing over time in wild-type controls (Fig. 2a).

The liver expresses two Akt isoforms (Akt1 and 2) and to better explore the role of PI3K-C2γ in Akt activation, the effect of PI3K-C2γ depletion on isoform-specific Akt phosphorylation was next examined using validated isoform-selective antibodies (Supplementary Fig. 2a). Unexpectedly, the lack of PI3K-C2γ in either livers (Fig. 2b) or isolated primary hepatocytes (Fig. 2c) did not affect the phosphorylation of Akt1, either at early or at late time points. On the contrary, the time course of Akt2 phosphorylation was significantly altered in insulin-stimulated *Pik3c2g*^*−/−*^ livers and primary hepatocytes (Fig. 2b,c). Similar to the observed profile of global Akt activation, phosphorylation of Akt2 at 5 min was comparable in both *Pik3c2g*^*−/−*^ and *Pik3c2g*^*+/+*^ samples. However, phosphorylated Akt2 rapidly declined in mutant livers that showed a significantly faster inactivation rate with a 50% reduction in Akt2 phosphorylation at 15 and 30 min (Fig. 2b,c). As shown in Supplementary Fig. 2b, mutant samples confirmed a reduced phosphorylation of Akt on Thr308/309 at later time points. Consistently, a significant reduction in the phosphorylation on Thr309 of Akt2 was detected in mutant livers at 30 min after insulin stimulation (Supplementary Fig. 2c). Analysis of insulin-evoked residual Akt phosphorylation of Ser473 of Akt1 in liver extracts of *Akt2*-deficient mice confirmed that Akt2 is the major hepatic isoform activated at 30 min after insulin stimulation (Fig. 2a and Supplementary Fig. 3a), thus indicating that the decrease in total pAkt observed in *Pik3c2g*^*−/−*^ mice is in line with a selective impairment of Akt2 activation at late time points. This result was clearly restricted to the liver of mutant mice, as the insulin-dependent Akt phosphorylation was normal in both skeletal muscles and adipose tissue (Supplementary Fig. 3b,c). Taken together, these findings indicate that PI3K-C2γ is not involved in acute insulin-dependent Akt activation but selectively supports prolonged phosphorylation of hepatic Akt2.

### PI3K-C2γ regulates a specific subset of Akt effectors

**Figure 3:**
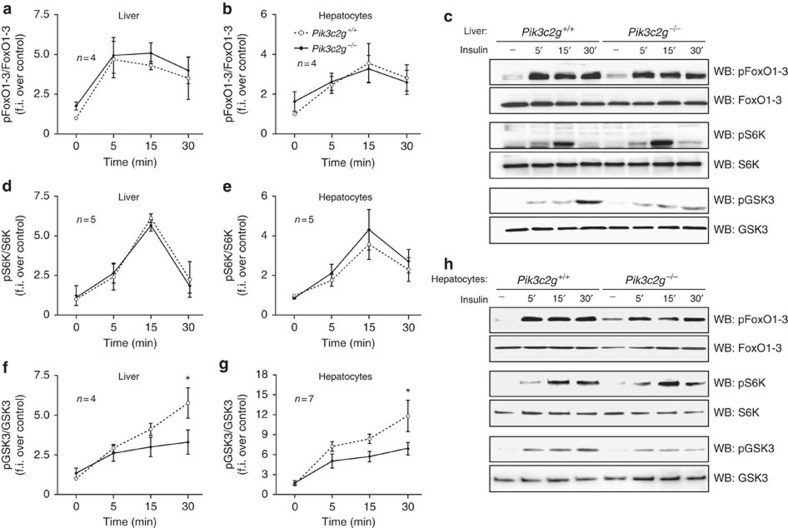
Loss of PI3K-C2γ impairs specific insulin responses downstream of Akt. (**a**, **b**) Analysis of FoxO1-3 phosphorylation in livers (**a**) and primary hepatocytes (**b**) from *Pik3c2g*^*+/+*^ and *Pik3c2g*^*−/−*^ mice stimulated with insulin (*n*=4 for each genotype). (**c**) Representative western blot (WB) of *n*=4, 5, 4 replicates showing FoxO1-3, S6K and GSK3 phosphorylation status, respectively, in livers from *Pik3c2g*^*+/+*^ and *Pik3c2g*^*−/−*^ mice, after the given times following intraperitoneal administration of insulin. (**d**, **e**) WB analysis of S6K phosphorylation in liver extracts (**d**) and primary hepatocytes (**e**) from *Pik3c2g*^*+/+*^ and *Pik3c2g*^*−/−*^ mice stimulated with insulin (*n*=5 for each genotype). (**f**, **g**) WB analysis of GSK3 phosphorylation in liver extracts (**f**) and primary hepatocytes (**g**) from *Pik3c2g*^*+/+*^ and *Pik3c2g*^*−/−*^ mice stimulated with insulin. Immunoblots are representative of four independent experiments (*n*=5 and 7 for each genotype, for liver and hepatocytes, respectively). (**h**) Representative WB analysis of *n*=4, 5, 7 replicates showing FoxO1-3, S6K and GSK phosphorylation status, respectively, before and after insulin stimulation of primary hepatocytes derived from *Pik3c2g*^*+/+*^ and *Pik3c2g*^*−/−*^ mice. Results represent mean±s.e.m of the number of replicates (*n*) reported in the figures. *P<0.05, **P<0.01 mutant versus the respective wild-type controls. *P* values were determined using two-way analysis of variance followed by Bonferroni *post-hoc* test. F.i., fold induction.

**Figure 4:**
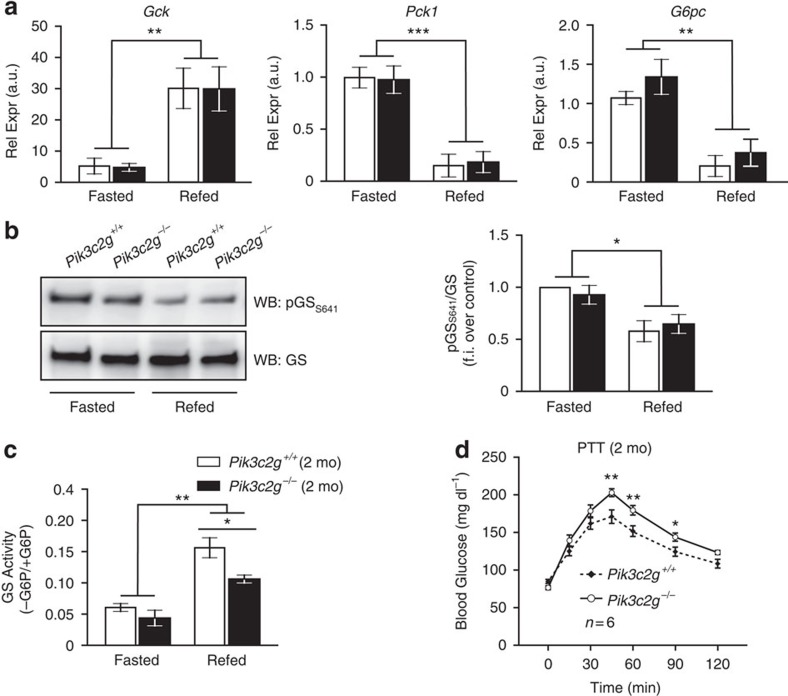
Loss of PI3K-C2γ impairs glycogen synthesis and hepatic glucose output control. (**a**) Real-time PCR analysis of mRNA levels of glucokinase (*Gck*), phosphoenolpyruvate carboxykinase (*Pck1*) and glucose-6-phosphatase catalytic subunit (*G6pc*) was performed from *Pik3c2g*^*+/+*^ and *Pik3c2g*^*−/−*^ liver extracts in overnight fasted (*n*=6 for each genotype) followed by 4 h refeeding (Refed, *n*=6 for each genotype) conditions. (**b**) Immunoblot for phosphorylated glycogen synthase (GS) in livers from fasted mice and after 1 h refeeding (left). Quantification of GS phosphorylation at Ser641 before and after refeeding is shown in the right panel. (**c**) Hepatic GS activity in fasting conditions or after 1 h refeeding (*n*=8 for each genotype/condition). Data are presented as ratio between sampled GS (-G6P) and allosterically activated total GS activity (+G6P). (**d**) Pyruvate challenge (PTT) of *Pik3c2g*^*+/+*^ and *Pik3c2g*^*−/−*^ mice. A bolus of 2 g kg^−1^ was administered intraperitoneally in 2-month (mo)-old mice fasted for 16 h, and the amount of blood glucose was measured at the indicated time points (*n*=6). Results represent mean±s.e.m for the given number (*n*) of replicates or mice. *P<0.05, **P<0.01, ***P<0.001 fasted versus refed or mutant versus the respective wild-type controls. *P* values were determined using one-way (**a**–**c**) or two-way analysis of variance followed by Bonferroni *post-hoc* test. WB, western blot.

The propagation of Akt-mediated signals involves the modulation of multiple direct and indirect downstream targets including forkhead box O (FoxO), S6K and glycogen synthase^29^. The potential impact of the altered Akt2 activation kinetics on the phosphorylation of such key downstream mediators was thus evaluated. In liver samples of insulin-stimulated mutant mice, the phosphorylation of the transcription factors FoxO1-3 was unaltered at all time-points assessed (Fig. 3a,c). Similar results were obtained studying the response in isolated hepatocytes (Fig. 3b,h). Likewise, S6K phosphorylation was unaltered both in livers after insulin administration *in vivo* and in primary hepatocytes (Fig. 3c,d,e,h), indicating that loss of PI3K-C2γ does not perturb activation of the mammalian target of rapamycin complex 1 (mTORC1) pathway. Conversely, in mutant liver and hepatocytes, phosphorylation of glycogen synthase kinase 3 (GSK3) on the Akt-dependent Ser9 slightly but significantly declined at later time points (Fig. 3c,f,g,h). Consistent with their normal FoxO1-3 activation status, mutant mice displayed no difference in FoxO-dependent expression of glucokinase (*Gck*), as well as gluconeogenic genes, such as phosphoenolpyruvate carboxykinase (P*ck1*) and glucose 6-phosphatase catalytic subunit (*G6*p*c*; Fig. 4a). Furthermore, the analysis of the effects of the loss of PI3K-C2γ on glycogen synthase phosphorylation failed to determine a significant alteration in GSK3-dependent GS phosphorylation in both fasted and refed livers (Fig. 4b), in line with a regulation of the enzyme in the liver independent of the phosphorylation of classical GSK3 sites^28^. Nonetheless, Akt2-mediated hepatic GS activation can occur independently of GSK3 (refs 27, 28) and, in agreement, the assessment of GS activity, in livers after overnight fasting followed by 1 h refeeding, showed that the loss of PI3K-C2γ caused a 30% decrease in GS activity (Fig. 4c). Overall, these results indicate that PI3K-C2γ specifically controls a subset of Akt2 substrates and that is critically involved in the activation of GS.

Given that normal regulation of gluconeogenic genes but the impaired activation of glycogen synthesis might increase the abundance of diffusible glucose within hepatocytes, mutant mice were challenged in a pyruvate-induced stimulation of glucose production in the liver. In this test, mutant mice showed a mild but significant increase of hepatic glucose output (Fig. 4d). As this could not be attributed to changes in the abundance of the liver glucose transporter GLUT2 (Supplementary Fig. 3d), the loss of PI3K-C2γ caused an impairment of the insulin-dependent hepatic glucose output inhibition likely due to decreased conversion of glucose into glycogen. This is consistent with studies on mice lacking Akt2 in the liver^27^,^28^ and further confirms PI3K-C2γ as a key determinant of hepatic Akt2 activation.

### Loss of insulin-dependent PtdIns(3,4)*P*_2_ in *Pik3c2g* ^*−/−*^ livers

**Figure 5:**
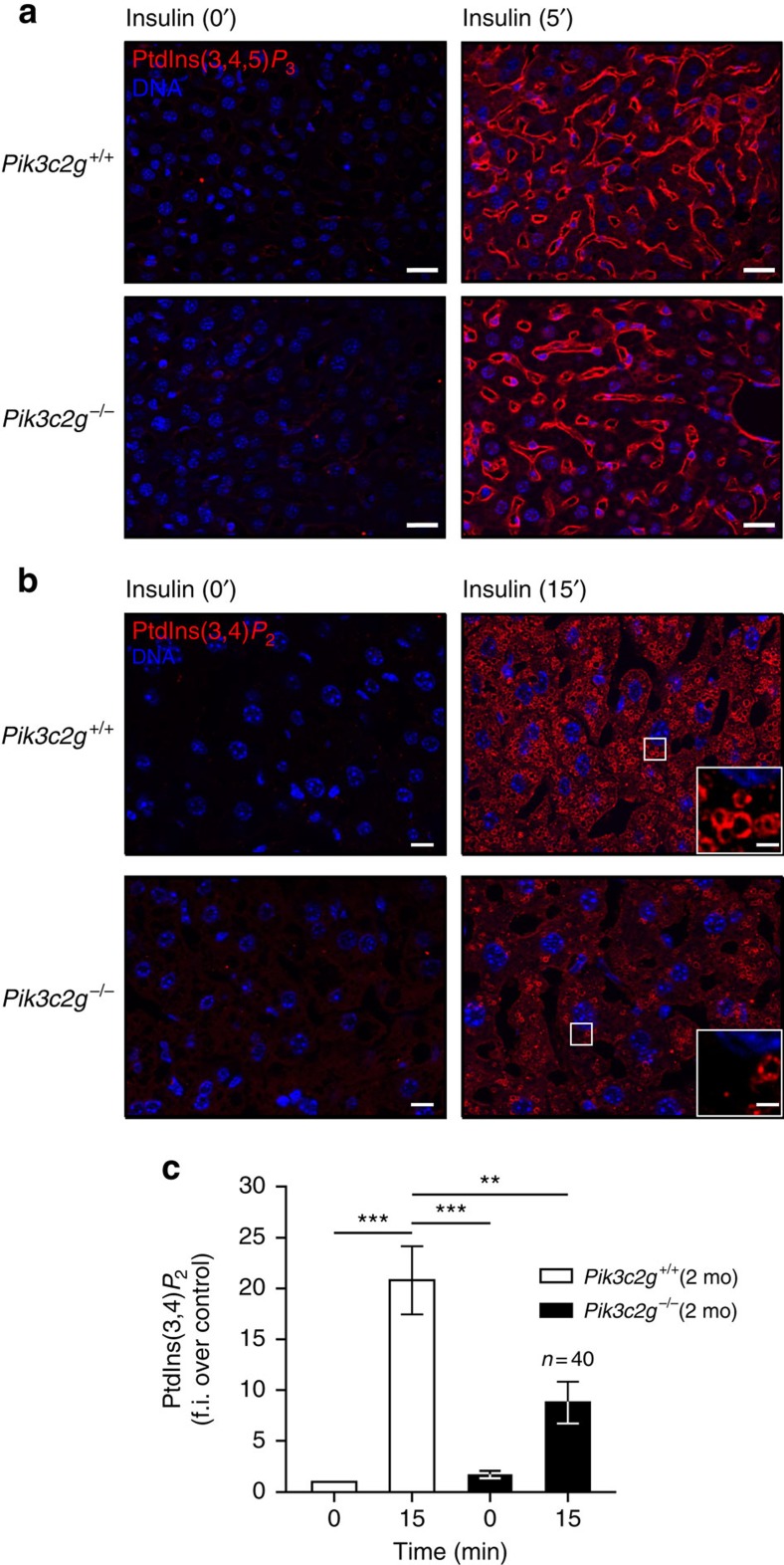
Reduced insulin-dependent PtdIns(3,4)*P*_2_ production in *Pik3c2g*^*−/−*^ livers. (**a**) Immunohistochemical detection of PtdIns(3,4,5)*P*_3_ in liver cryosections obtained from fasted *Pik3c2g*^*+/+*^ and *Pik3c2g*^*−/−*^ 2-month (mo)-old mice before (insulin 0′) and 5 min after insulin stimulation (insulin 5′). Images are representative of similar results obtained in seven independent experiments. Scale bar, 20 μm. (**b**) Immunohistochemical detection of PtdIns(3,4)*P*_2_ in liver cryosections obtained from fasted *Pik3c2g*^*+/+*^ and *Pik3c2g*^*−/−*^ 2-mo-old mice before (insulin 0′) and 15 min after insulin stimulation (insulin 15′). Images are representative of similar results obtained in eight independent experiments per condition and genotype. Scale bar, 10 μm. Insets indicate vesicular accumulation of PtdIns(3,4)*P*_2_. Scale bar, 2 μm. (**c**) Quantification of PtdIns(3,4)*P*_2_ immunofluorescence obtained from ten independent images (per genotype) of the experiments shown in **b** (*n*=40). Results represent mean±s.e.m for the given number (*n*) of images. *P<0.05, **P<0.01, ***P<0.001 fasted versus insulin-stimulated or mutant versus the respective wild-type controls. *P* values were determined using one-way analysis of variance followed by Bonferroni *post-hoc* test. F.i., fold induction.

Insulin-stimulated Akt activation requires the presence of the two PI3K products phosphatidylinositol-3,4,5-trisphosphate (PtdIns(3,4,5)*P*_3_) and phosphatidylinositol-3,4-bisphosphate (PtdIns(3,4)*P*_2_), membrane lipids that constitute docking sites for PH domain-mediated protein anchoring required for Akt activating phosphorylation events^30^. Although PtdIns(3,4,5)*P*_3_ is a potent Akt activator, class II PI3Ks are usually reported not to produce this mediator *in vitro* or *in vivo*^31^. To confirm this hypothesis, PtdIns(3,4,5)*P*_3_ levels were assayed *in vivo* by immunofluorescence, using specific antibodies to stain sections of livers, before and after insulin stimulation. Although staining of baseline PtdIns(3,4,5)*P*_3_ in fasted *Pik3c2g*^*−/−*^ and *Pik3c2g*^*+/+*^ mice was, as expected, very low, production of this mediator, 5 min after the insulin bolus, equally increased in hepatocyte sinusoidal plasma membranes of both genotypes, hence supporting the view that PI3K-C2γ is not involved in PtdIns(3,4,5)*P*_3_ production *in vivo* (Fig. 5a). The two genotypes also showed a similarly negligible baseline of PtdIns(3,4)*P*_2_ (Fig. 5b). However, 15 min after insulin administration, a clear PtdIns(3,4)*P*_2_ signal was detectable in wild-type liver samples and localized in intracellular vesicular structures. Although the number of Rab5-positive vesicles was comparable in the two genotypes (Supplementary Fig. 4a), the number of PtdIns(3,4)*P*_2_-positive vesicles appeared overtly decreased in *Pik3c2g*^*−/−*^ liver samples (Fig. 5b). Quantification of fluorescence showed a significant fourfold reduction in mutant livers (Fig. 5c). To exclude the possibility that, in our experimental conditions, PtdIns(3,4)*P*_2_ staining was unspecific, competition experiments were performed by pre-incubating the PtdIns(3,4)*P*_2_ antibody on liposomes containing synthetic PtdIns(3,4)*P*_2_. This procedure abolished the signal on liver sections of wild-type mice stimulated with insulin (Supplementary Fig. 4b). On the contrary, pre-incubation of the PtdIns(3,4)*P*_2_ antibody on liposomes containing the unrelated control lipid PtdIns(4,5)P_2_ did not modify the staining pattern of wild-type control sections (Supplementary Fig. 4b). In further agreement, immunodetection of PtdIns(3,4)*P*_2_ in wild-type and mutant primary hepatocytes showed that 15 min after insulin stimulation the number of PtdIns(3,4)*P*_2_-positive vesicles was significantly reduced in PI3K-C2γ-deficient cells (Supplementary Fig. 5). These results overall demonstrate that, in response to insulin, PI3K-C2γ is crucially involved in the generation a PtdIns(3,4)*P*_2_ pool spatially localized on endosomal membranes.

### Insulin triggers PI3K-C2γ localization on EEs

**Figure 6:**
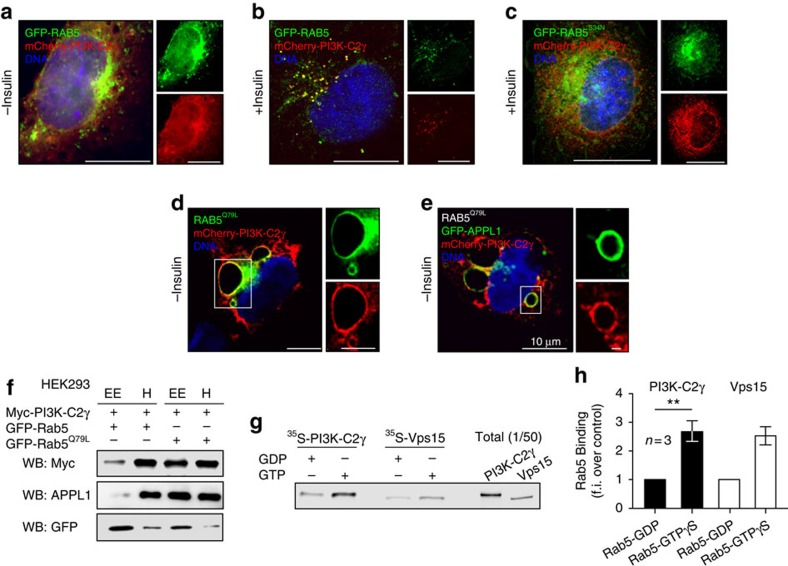
PI3K-C2γ is a Rab5 effector in early endosomes (EEs). (**a**, **b**) Co-localization of PI3K-C2γ and Rab5 before (**a**) or after (**b**) insulin stimulation by confocal analysis. COS-7 cells were transfected with mCherry-PI3K-C2γ (red) and GFP-Rab5 (green), serum starved for 5 h and incubated without (**a**) or with insulin for 10 min at 37 °C (**b**). mCherry-PI3K-C2γ and GFP-Rab5 co-localized in punctate structures, in the presence of insulin (**b**). Scale bar, 10 μm. (**c**) Cytosolic localization by confocal analysis of mCherry-PI3K-C2γ (red) in dominant negative GFP-Rab5^S34N^ (green) transfected and serum-starved COS-7 cells. Scale bar, 10 μm. (**d**) Co-localization of GFP-Rab5^Q79L^ (green) and mCherry-PI3K-C2γ (red) in transfected COS-7 cells. Scale bar, 10 μm. (**e**) Co-localization by confocal analysis of GFP-APPL1 (green) and mCherry-PI3K-C2γ (red) in Rab5^Q79L^-transfected COS-7 cells. Scale bar, 10 μm. (**f**) Western blot (WB) analysis of Myc-PI3K-C2γ and GFP-Rab5 or GFP-Rab5^Q79L^ distribution in different subcellular fractions (H: cytosolic/heavy membranes). (**g**) PI3K-C2γ binds directly to activated Rab5A. GST-Rab5A beads loaded with either GDP or GTPγS were incubated with [^35^S]-labelled *in vitro* translated PI3K-C2γ. The beads were washed and analysed by SDS–PAGE and autoradiography. Vps15, which is known to bind to GST-Rab5A in a GTP-dependent manner, was used as a control. (**h**) Quantitative densitometry of the WB in **g** (*n*=3). Results represent mean±s.e.m. **P<0.01. *P* values were determined using one-way analysis of variance followed by Bonferroni *post-hoc* test. F.i., fold induction.

The nature of the PI3K-C2γ-positive endosomes was explored in HEK293 subcellular fraction expressing a Myc-tagged PI3K-C2γ, as specific antibodies against murine PI3K-C2γ were not available, even despite extensive immunization attempts with different peptides and fusion proteins. Cell fractionation showed that, before and after insulin stimulation, Myc-PI3K-C2γ was undetectable in Rab7^+^/APPL1^*−*^ late endosomes (LEs; Supplementary Fig. 6a). Conversely, Myc-PI3K-C2γ was found on Rab5^+^/APPL1^+^ EEs in basal conditions as well as in response to insulin (Supplementary Fig. 6b). The amount of Myc-PI3K-C2γ and APPL1 in Rab5^+^ EEs was significantly higher after insulin stimulation than in resting conditions, suggesting an insulin-dependent recruitment of PI3K-C2γ to this specific cellular compartment (Supplementary Fig. 6c). In agreement, a mCherry-PI3K-C2γ fluorescent reporter was dispersed in resting COS7 cells (Fig. 6a) but, upon insulin stimulation, it co-localized with GFP-Rab5 on Rab5^+^ EEs (Fig. 6b). On the other hand, the expression of a dominant negative GFP-Rab5^S34N^ led to a diffuse cytoplasmic distribution of mCherry-PI3K-C2γ and abrogated the insulin-dependent PI3K-C2γ/Rab5 co-localization (Fig. 6c). Similar results were obtained in wild-type primary hepatocytes transfected to express a Myc-tagged PI3K-C2γ and co-labelled with Myc and Rab5 antibodies (Supplementary Fig. 7). In agreement with Rab5-dependent endosomal recruitment of PI3K-C2γ, expression of a constitutively active GFP-Rab5^Q79L^ led to insulin independent, constitutive localization of mCherry-PI3K-C2γ to the giant Rab5^+^ endosomes induced by the expression of the Rab5 mutant (Fig. 6d). Similarly, expression of an untagged Rab5^Q79L^ caused the co-localization of mCherry-PI3K-C2γ and APPL1^+^ to typical Rab5^Q79L^-dependent endosomes (Fig. 6e). Next, cell fractionation studies were repeated after transfection of either GFP-Rab5 or GFP-Rab5^Q79L^. As shown in Fig. 6f, Rab5^Q79L^ but not Rab5 induced the enrichment of Myc-PI3K-C2γ in Rab5^+^/APPL1^+^ early endosomal fraction.

To test whether PI3K-C2γ is a direct Rab5 effector, binding of *in vitro* translated ^35^S-labelled PI3K-C2γ to purified Rab5 was assessed in pull-down experiments with inactive GDP-bound and active GTP-γS-bound Rab5. As indicated in Fig. 6g,h, labelled PI3K-C2γ directly bound Rab5-containing beads and this interaction was significantly enhanced by the presence of active Rab5 to an extent similar to the known Rab5 effector Vps15 (ref. 32). Altogether, these data indicate that PI3K-C2γ directly acts downstream of Rab5 during the process of insulin receptor internalization in EEs.

### PI3K-C2γ loss impairs Akt phosphorylation on EEs

**Figure 7:**
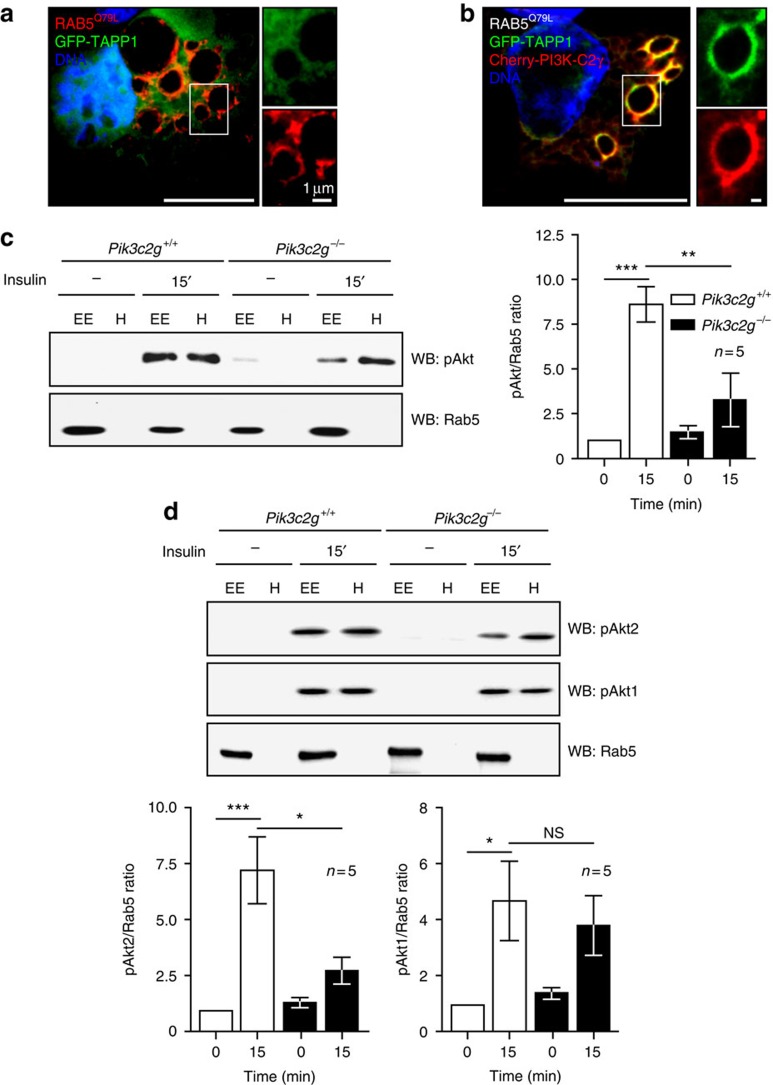
PI3K-C2γ is required for Akt activation on early endosomes (EEs) *in vivo*. (**a**) Confocal imaging of a COS-7 cell transfected with Rab5^Q79L^ and GFP-TAPP1 (green). Immunostaining for Rab5 is shown in red. (**b**) Co-localization by confocal imaging of GFP-TAPP1 (green) and mCherry-PI3K-C2γ (red) in Rab5^Q79L^-transfected COS-7 cells. Scale bar, 10 μm. (**c**) Analysis of Akt phosphorylation in the EE fraction obtained from *Pik3c2g*^+/+^ and *Pik3c2g*^*−/−*^ livers before and 15 min after insulin injection; subcellular fractions enriched in EEs and heavy membranes (H) were resolved by SDS–PAGE and subjected to Western blot (WB) analysis with the indicated antibodies. Immunoblots are representative of five independent experiments. The right panel shows the quantification of the relative levels of pAkt detected in the EE fraction. (**d**) Analysis of Akt1 and Akt2 phosphorylation in the EE fraction as in **c**. Immunoblots are representative of five independent experiments. The panels below show the quantification of the relative levels of pAkt1 and pAkt2 detected in the EE fraction. Results represent mean±s.e.m for the given number (*n*) of images. *P<0.05, **P<0.01, ***P<0.001 starved versus insulin-stimulated and mutant versus the respective wild-type controls. *P* values were determined using one way analysis of variance followed by Bonferroni *post-hoc* test. NS, not significant.

Next, to test if the Rab5-dependent localization of PI3K-C2γ to EEs was functionally relevant, constitutively active Rab5 (Rab5^Q79L^) was transfected with a GFP-TAPP1 PtdIns(3,4)*P*_2_ fluorescent probe in the presence or absence of mCherry-PI3K-C2γ. As shown in Fig. 7a, the expression of Rab5^Q79L^ induced the presence of giant endosomes but was not sufficient to induce endosomal accumulation of PtdIns(3,4)*P*_2_ in cell lines expressing undetectable levels of endogenous PI3K-C2γ-encoding mRNA like COS7 cells. On the other hand, the concomitant expression of mCherry-PI3K-C2γ in the same Rab5^Q79L+^/GFP-TAPP1^+^ COS7 promoted the targeting to giant endosomes of the GFP-TAPP1 PtdIns(3,4)*P*_2_ fluorescent probe (Fig. 7b). This demonstrates that PI3K-C2γ is a downstream effector of Rab5 inducing PtdIns(3,4)*P*_2_ accumulation on EEs.

To further assess the role of PI3K-C2γ-dependent PtdIns(3,4)*P*_2_ production on EEs on Akt phosphorylation, cell fractionation studies were performed in intact livers of wild-type and mutant mice before and 15 min after insulin stimulation. In response to insulin administration, the Rab5-positive endosomal fraction of wild-type samples displayed a marked phosphorylation of Akt on Ser473 (Fig. 7c). On the contrary, in the corresponding Rab5-positive fraction from *Pik3c2g*^*−/−*^ liver samples, phosphorylation of Akt at 15 min after insulin administration appeared significantly reduced, thus supporting the hypothesis that PI3K-C2γ plays a crucial role in regulating the Rab5-dependent endocytic Akt activation. In keeping with a preferential role of PI3K-C2γ in the activation of Akt2 in Rab5^+^ EEs, antibodies specifically distinguishing the phosphorylation of Ser473 of either Akt1 or Ak2 showed that Akt2 but not Akt1 phosphorylation is significantly decreased in EEs of insulin-stimulated *Pik3c2g*^*−/−*^ livers (Fig. 7d).

### Increased obesity and fatty liver in HFD-fed *Pik3c2g* ^*−/−*^ mice

These results indicate that PI3K-C2γ represents a branch point in endosomal insulin signalling, balancing fat and glucose metabolism, and suggest that this enzyme might be required to maintain homeostasis in response to stress conditions. To assess this possibility, the potential metabolic derangement occurring with age and in response to a diabetogenic high-fat diet was studied.

**Figure 8:**
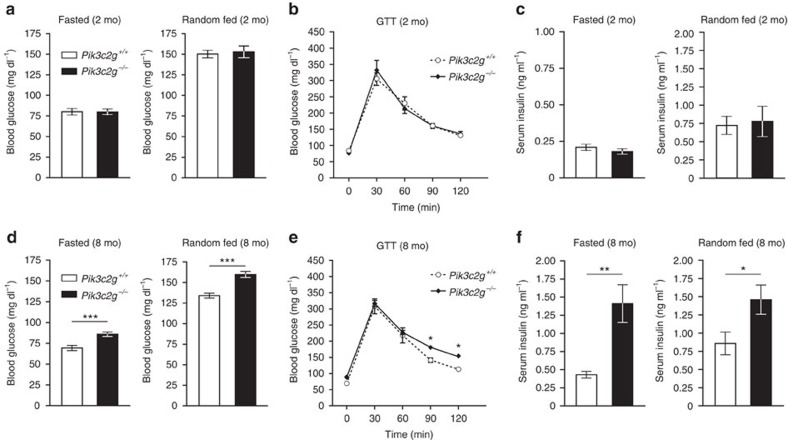
PI3K-C2γ-deficient mice develop glucose intolerance with age. (**a**) Fasted and fed blood glucose levels in wild-type (*Pik3c2g*^*+/+*^) and knockout mice (*Pik3c2g*^*−/−*^) measured at 2 months (mo) of age (*n*=14 and *n*=9, respectively). (**b**) Glucose tolerance test (GTT) in 2-mo-old *Pik3c2g*^*+/+*^ (open circles) and *Pik3c2g*^*−/−*^ (filled diamonds) mice (*n*=8 and *n*=9, respectively). (**c**) Serum insulin levels measured in *Pik3c2g*^*+/+*^ and *Pik3c2g*^*−/−*^ mice at 2 mo of age in fasted (*n*=7 and *n*=4, respectively) and fed conditions (*n*=11 and *n*=8, respectively). (**d**) Fasted and fed blood glucose levels in 8-mo-old *Pik3c2g*^*+/+*^ and *Pik3c2g*^*−/−*^ mice (*n*=9 and *n*=12, respectively). (**e**) GTT in 8-mo-old *Pik3c2g*^*+/+*^ and *Pik3c2g*^*−/−*^ mice (*n*=5 and *n*=13, respectively). (**f**) Serum insulin levels measured in 8-mo-old *Pik3c2g*^*+/+*^ and *Pik3c2g*^*−/−*^ mice in fasted (*n*=6 and *n*=4, respectively) and fed conditions (*n*=7 and *n*=4, respectively). Results represent mean±s.e.m for the given number (*n*). *P<0.05, **P<0.01, ***P<0.001 mutant versus the respective wild-type controls. *P* values were determined using Student’s *t*-test (**a**, **c**, **d**, **f**) or one-way analysis of variance followed by Bonferroni *post-hoc* test (**b**, **e**).

First, the circulating levels of glucose were measured in 2-month-old *Pik3c2g*^*−/−*^ and *Pik3c2g*^*+/+*^ mice. At this age, the ablation of *Pik3c2g* did not affect either blood glucose or insulin levels, in fasting as well as in fed conditions (Fig. 8a). Similarly, glucose reduction rates in a glucose tolerance test (GTT) appeared comparable in both homozygotes and 2-month-old wild-type controls (Fig. 8b). In agreement with normal glucose tolerance, 2-month-old *Pik3c2g*^*−/−*^ mice did not show any change in serum insulin levels (Fig. 8c). On the contrary, mutant mice had increased circulating glucose levels during aging; 8-month-old *Pik3c2g*^*−/−*^ mice showed a mild but significant blood glucose increase, in both fasted and fed conditions (Fig. 8d). Together with the presence of glucose intolerance (Fig. 8e), this defect suggests that PI3K-C2γ ablation might contribute to the onset of age-dependent insulin resistance. In support of this view, 8-month-old mutant mice displayed about four- and twofold increases in serum insulin under fasted and fed conditions, respectively (Fig. 8f).

**Figure 9:**
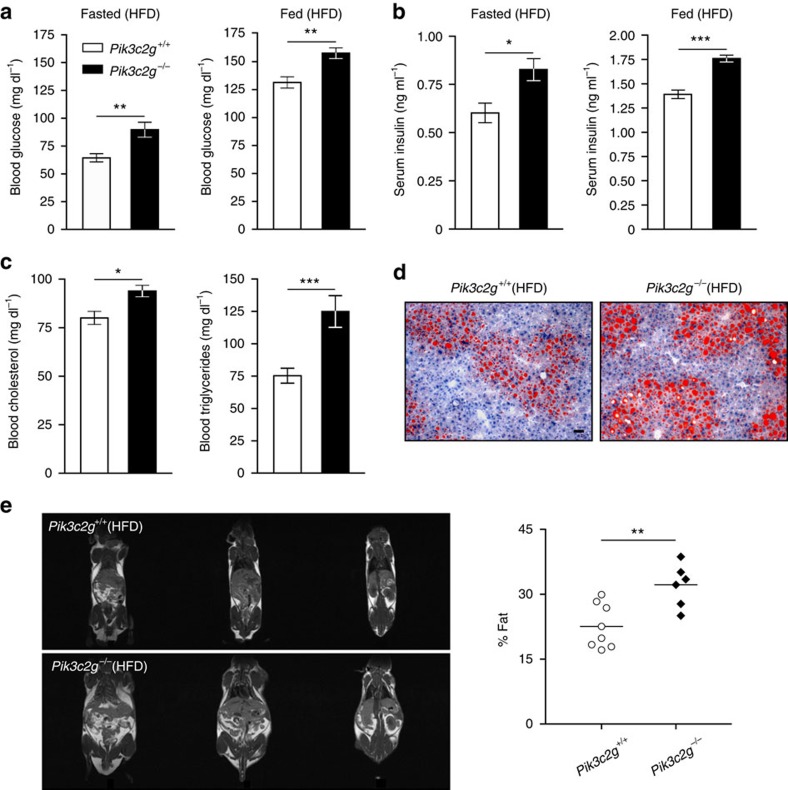
Insulin resistance and dysfunctional lipid metabolism in *Pik3c2g*^*−/−*^ mice after 16 weeks of high-fat diet (HFD). (**a**) Blood glucose levels in *Pik3c2g*^*+/+*^ and *Pik3c2g*^*−/−*^ measured after 16 weeks of feeding with HFD, in fasted (*n*=15 and *n*=8, respectively) and fed conditions (*n*=13 and *n*=8, respectively). (**b**) Serum insulin levels in HFD-fed *Pik3c2g*^*+/+*^ and *Pik3c2g*^*−/−*^ mice were measured in fasted (*n*=9 and *n*=6, respectively) and fed conditions (*n*=7 and *n*=6, respectively). (**c**) Assessment of blood cholesterol (*n*=11 and *n*=5, respectively) and triglycerides (*n*=12 and *n*=6, respectively) in *Pik3c2g*^*+/+*^ and *Pik3c2g*^*−/−*^ mice fed HFD for 16 weeks. (**d**) Representative images of Oil red O-stained liver sections, obtained from HFD-fed *Pik3c2g*^*+/+*^ and *Pik3c2g*^*−/−*^ mice (*n*=10 and *n*=6, respectively). Scale bar, 40 μm. (**e**) Representative Coronal T_1-weighted_ spin-echo whole-body magnetic resonance imaging (MRI) scans of HFD-fed *Pik3c2g*^*+/+*^ and *Pik3c2g*^*−/−*^ mice (left) and fat mass quantification (right). Results in **a**–**c** represent mean±s.e.m. Quantification of body fat percentage (**e**) was performed by segmenting images into three intensities using a k-means clustering algorithm, with the volumes of highest intensities corresponding to adipose regions. The horizontal bar represents the mean value. *P<0.05, **P<0.01, ***P<0.001 mutant versus the respective wild-type controls. *P* values were determined using Student’s *t*-test.

To further check if this propensity to insulin resistance with age could be accelerated by diet, 1-month-old *Pik3c2g*^*−/−*^ mutants and control mice were fed with a fat-rich diet (high-fat diet (HFD), 60% kcals derived from fat) for 4 months. The impact of such treatment on glucose homeostatic responses was next evaluated. Glycaemia, in fasted and fed conditions, appeared 30% and 17% higher in *Pik3c2g*^*−/−*^ than in wild-type controls, respectively (Fig. 9a). This was accompanied by significant 21% and 19% increases in insulinemia in both conditions (Fig. 9b) that was comparable to that of aged mutant homozygotes (Fig. 8f). In line with this observation, HFD-fed *Pik3c2g*^*−/−*^ mice displayed a mild but significant impairment in glucose tolerance, whereas age-matched controls on normal diet were still normal (Supplementary Fig. 8a,b). Furthermore, HFD-fed, but not chow-diet fed, *Pik3c2g*^*−/−*^ mice showed increased body weight gain compared with control mice (Supplementary Fig. 8c,d), and after 4 months of treatment, they were 18% heavier than their wild-type counterparts (39.26±6.50 g in *Pik3c2g*^*−/−*^ versus 33.35±4.03 g in *Pik3c2g*^*+/+*^; *P*<0.01 (by Student’s *t*-test)). This body weight increase was associated with dyslipidemia, increased plasma concentrations of cholesterol and triglycerides (Fig. 9c). Consistently, histopathological assessment of livers derived from HFD-fed mutant mice showed increased lipid droplets deposition, a hallmark of hepatic triglyceride accumulation (Fig. 9d), whereas livers of mice on normal chow remained unaffected (Supplementary Fig. 8e). In agreement with these observations, the hepatic mRNA levels of lipogenic genes such as Sterol regulatory element-binding protein-1 (*Srebp-1*), Steaoryl-CoA desaturase-1 (*Scd-1*) and fatty acid synthase (*Fasn*) were 60%, 42% and 40% higher in *Pik3c2g*^*−/−*^ mice than in wild-type controls, respectively (Supplementary Fig. 8f). Conversely, genes promoting fatty acid catabolism such as PPARγ coactivator-1α (P*gc1a*) and β (P*gc1b*) were 63% and 64% lower in *Pik3c2g*^*−/−*^ mice, respectively (Supplementary Fig. 8g). Furthermore, HFD-fed mutant mice showed defective hepatic glycogen accumulation (Supplementary Fig. 8h). In line with abnormal glucose homeostatic responses and with increased liver-mediated triglyceride production, epididymal fat from HFD-fed mice was significantly heavier in mutant mice than in wild-type controls (0.77±0.16 g versus 0.38±0.05 g; *P*<0.05 (Student’s *t* test)). This was further confirmed by the analysis of the percentage of body fat and lean mass by magnetic resonance imaging (MRI). This analysis evidenced a strikingly higher fat mass in HFD-fed *Pik3c2g*^*−/−*^ than in *Pik3c2g*^*+/+*^ mice (Fig. 9e), further confirming *Pik3c2g* as a gene controlling insulin sensitivity, glucose homeostasis and adiposity.

## Discussion

**Figure 10:**
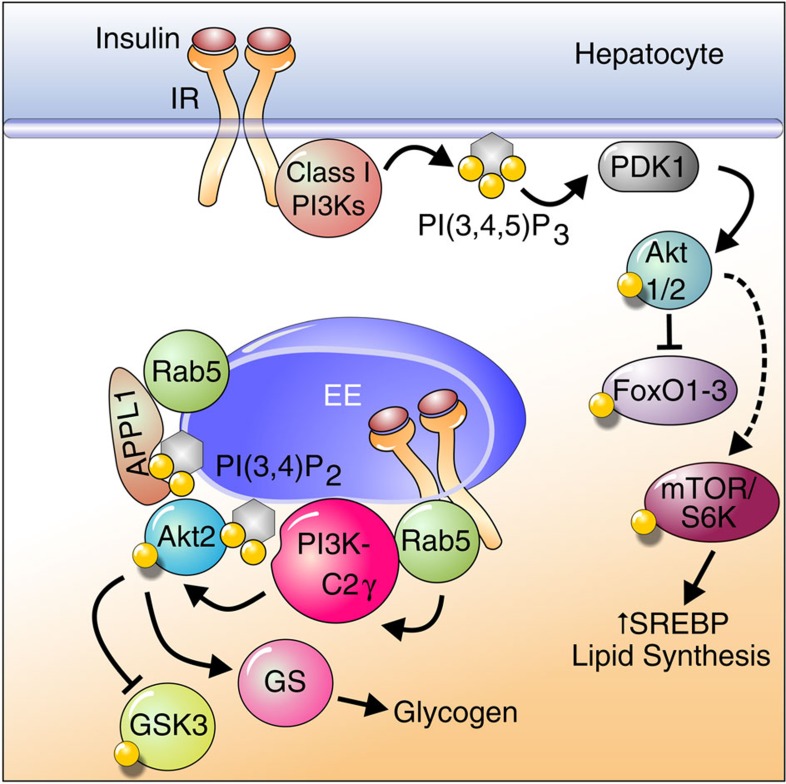
Scheme of action of liver PI3K-C2γ. Insulin receptor (IR) stimulation of hepatocytes triggers Akt phosphorylation through the classical PI3K pathway and stimulates mTOR/S6K-dependent regulation of lipid synthesis. However, Rab5-dependent PI3K-C2γ activation is responsible for the production of a pool of PtdIns(3,4)P2 at early endosomes (EEs) that specifically activates Akt2 and triggers GS synthase/glycogen production as well as GSK3 phosphorylation.

Although the specific function of PI3K-C2γ has long remained unexplored, our study provides evidence that this enzyme is critically required for delayed and sustained Akt2 activation downstream of insulin stimulation, and is necessary for the fine tuning of metabolic responses in the liver (Fig. 10).

Evidence for a role of PI3K-C2γ in the control of insulin metabolism emerged from the observation that *Pik3c2g*^*−/−*^ mice develop age-dependent glucose intolerance and marked hyperinsulinemia. Although this phenotype peaked in older animals, less pronounced impaired insulin sensitivity was already present in 2-month-old PI3K-C2γ-deficient mice that were characterized by reduced GS activation after refeeding and consequent decreased hepatic glycogen accumulation. Such a condition can be sufficient to trigger insulin resistance, as mice with heterozygous loss of protein targeting to glycogen, a scaffold protein involved in glycogen synthesis, show significant reduction in liver glycogen and an attenuated insulin receptor signalling^26^. Nonetheless, the mild insulin resistance detected in PI3K-C2γ-deficient mice was likely due to impaired inhibition of hepatic glucose output as suggested by the results of the pyruvate challenge. This effect might seem in contrast with the observed normal regulation of gluconeogenetic genes like *Pck1*. However, such findings are in line with what was previously reported in mice with liver-specific ablation of Akt2; these mice show normal *Pck1* transcriptional regulation but, due to impaired GS activation, they fail to induce rapid glucose diversion into glycogen^27^ and they develop insulin resistance^28^. Given that hepatic GS is mainly driven by an insulin-dependent glucose-6-phosphate-mediated allosteric activation^33^, our results further confirm Akt2 activation as a key insulin-evoked event redirecting glucose-6-phosphate to glycogen and required to effectively suppress glucose output^28^.

Consistent with these observations, the loss of PI3K-C2γ specifically reduced delayed and sustained insulin-dependent Akt2 phosphorylation. This isoform-selective class II PI3K-mediated Akt2 modulation indicates the existence of a so far unforeseen signalling process involving a specialized interplay between selected PI3K and Akt isoenzymes. Similarly, the absence of PI3K-C2γ appeared to specifically impact on GS activation, while leaving unaffected the modulation of other Akt effectors, such as the mTORC1/S6K axis and the FoxO1-3 transcription factors. In support to this view, the lack of PI3K-C2γ maintained an intact insulin/mTORC1-dependent *Srebp* upregulation as well as FoxO1-3-mediated transcriptional activation of genes controlling gluconeogenesis.

Despite such effects, younger mutant mice maintained normoglycemia and unaltered body weight. This likely occurred in response to the activation of compensatory processes: for example, *Pik3c2g*^*−/−*^ mice showed increased serum triglycerides and abnormally enlarged adipocytes starting from 2 months of age, thus indicating that storage of calories-rich molecules is diverted from glycogen to triglycerides. This situation might mimic the classical overfeeding response where an excess of glucose in a context of saturated glycogen storage causes the surplus glucose to be converted by the liver into fat that is subsequently stored in adipose tissue^34^. This is consistent with the observation that reduced insulin receptor function frequently leads to decreased liver mass and increased fat deposits^35^,^36^,^37^.

The bifurcation of insulin signalling into PI3K-C2γ-independent signalling versus PI3K-C2γ-dependent prolonged activation of Akt2 suggests the involvement of a spatially restricted subcellular compartmentalization. In agreement with this model, our studies in insulin-stimulated livers showed that, in the absence of PI3K-C2γ, a specific pool of endosomal PtdIns(3,4)*P*_2_ is severely reduced, thus supporting a role for PI3K-C2γ in spatial and temporal regulation of insulin signalling^38^,^39^,^40^,^41^. Within the first 5 min after insulin receptor activation, an acute wave of Akt phosphorylation occurs at the plasma membrane in response to a spike of PtdIns(3,4,5)*P*_3_, mainly produced by PI3Kα^3^. Although PtdIns(3,4,5)*P*_3_ is then rapidly removed by the phosphatases phosphatase and tensin homolog (PTEN) and SH2 domain-containing inositol 5'-phosphatase (SHIP)^42^, eliminating the phosphate on position 3 and 5, respectively, persistence of Akt phosphorylation is supported by PI3K pathway induction following endocytic internalization of the active insulin receptor complex^6^. Interestingly, studies on the ubiquitously expressed inositol polyphosphate 4-phosphatase A, which dephosphorylates position 4 of the inositol ring and acts on endosomes, show that this process relies on PtdIns(3,4)*P*_2_ rather than on PtdIns(3,4,5)*P*_3_. Indeed, inositol polyphosphate 4-phosphatase A depletion from EEs causes severe prolongation of Akt phosphorylation after growth factor stimulation^13^,^43^. The rapid action of this phosphatase also implies that the endosomal pool of PtdIns(3,4)*P*_2_ is short lived and, in line with this view, an HPLC analysis in wild-type and *Pik3c2g*^*−/−*^ primary hepatocytes failed to provide PtdIns(3,4)*P*_2_ in amounts amenable to quantification. Although a plethora of lipid kinases and phosphatase can contribute to PtdIns(3,4)*P*_2_ accumulation in endosomes^42^, our results point to a specifically localized function of PI3K-C2γ in the concomitant production of PtdIns(3,4)*P*_2_ and isoform-selective Akt2 activation. This is likely orchestrated by Rab5, a critical controller of endosomal system organization in the liver^44^ that is activated in insulin-stimulated cells^45^. Our subcellular localization studies showing that insulin induces the co-localization of PI3K-C2γ and Rab5 in vesicles corroborate this view. Furthermore, the finding that PI3K-C2γ directly binds Rab5-GTP together with the ability of a constitutively active Rab5^Q79L^ mutant to induce PI3K-C2γ localization as well as PI3K-C2γ-dependent PtdIns(3,4)*P*_2_ accumulation on EEs, support the view that PI3K-C2γ is a direct Rab5 effector. As Rab5 can promote Akt activation^5^, our results indicate that PtdIns(3,4)*P*_2_ produced by PI3K-C2γ plays a role in this process. Rab5 contributes to the selective activation of Akt2 through the endosomal targeting of the adaptor protein APPL1 (refs 46, 47), which promotes the preferential association/activation with this specific Akt isoform^6^,^48^,^49^. Our observations that PtdIns(3,4)*P*_2_ co-localizes with PI3K-C2γ in response to Rab5 activation support a model where PI3K-C2γ is critically required for the production of a lipid-activating Akt2 on EEs.

The ultimate outcome of the loss of PI3K-C2γ, together with the reduction in prolonged Akt2 activation, was insulin resistance and adiposity. This was particularly evident in response to high-fat diet, as PI3K-C2γ-deficient mice developed insulin resistance, dyslipidemia, fat mass increase and fatty liver. These conditions might be explained by the upregulation of the transcription factor Srebp1 and the concomitant downregulation of hepatic expression of β-oxidative enzymes (PGC1α and β). These results are in line with the finding that patients with severe insulin resistance linked to an Akt2 point mutation develop increased *de novo* lipogenesis and fatty liver^50^. Nonetheless, the *Srebp* transcriptional upregulation found in PI3K-C2γ-deficient mice appears in contrast with what was observed in liver-specific Akt2-null mouse mutants^51^. This discrepancy might lie in the partial Akt2 signalling impairment in *Pik3c2g*^*−/−*^mice, which did not affect the acute activation of Akt2 at the plasma membrane. This residual Akt2 activation appears sufficient to drive the increased pathway activation typically observed in the presence of high serum insulin levels.

Overall, our data indicate that loss of PI3K-C2γ induces the development of a condition frequently preceding the onset of type II diabetes mellitus. This is consistent with the report of the association between a nucleotide polymorphism in the human *PIK3C2G* gene and type II diabetes mellitus^25^. Therefore, our results linking PI3K-C2γ with Akt2 and metabolic control in the liver provide a molecular mechanism for this association and suggest this PI3K-C2γ as a key element protecting from age-associated and diet-related insulin resistance.

## Methods

### Generation of PI3K-C2γ knockout mice

We created a knockout construct by insertion of *lacZ* reporter gene into the *Pik3c2g* exon1 downstream to the ATG codon *Pik3c2g* by homologous recombination strategy. The targeting construct following *lacZ* gene contained a neomycin-resistant cassette, thus permitting the G418 selection of transfected ES cells. The specific insertion in *Pik3c2g* locus was determined by Southern blot analysis and one of the positive ES clones was injected in blastocysts. The chimeric mice obtained were mated with C57BL/6 mice. The heterozygous *Pik3c2g*^*+/lacZ*^ mice were mated to obtain knockout littermates. Akt2-deficient mice^52^ were purchased from Jackson Laboratories.

### Mice and genotype analysis

For all analysis, mice were maintained on a 129Sv-C57BL/6 mixed background and were handled according to institutional animal welfare guidelines and legislation, as approved by the local Animal Ethics Committee (Comitato di Bioetica e Valutazione, Torino, Italy). Phenotypes were confirmed in both male and females either at the specified age. Genotyping was performed by PCR using genomic DNA isolated by tails. The presence of the wild-type allele was detected using the following primers: wtc2gfor 5′-AGTGAGCAACCCAAGCACTTGC-3′ and wtkoc2grev 5′-ACAGCAGGATTAAAACCAATGGCTG-3′. For the detection of the mutant allele with insertion of neomycin cassette, the following primers were employed: koc2gfor 5′-TATCAGGACATAGCGTTGGCTACCCGTG-3′ and wtkoc2grev 5′-ACAGCAGGATTAAAACCAATGGCTG-3′.

### Plasmid vectors and constructs

Myc-tagged and mCherry-tagged PI3K-C2γ constructs were made starting from I.M.A.G.E. full-length cDNA clone of human *PIK3C2G* (ID 40146308; Source BioScience LifeSciences). N-terminal Myc-tagged PI3K-C2γ cDNA was inserted into the mammalian expression vector pcDNA3.1 (Invitrogen). mCherry sequence was inserted at N-terminus of PI3K-C2γ cDNA into pcDNA3.1 plasmid.

### Reverse transcription–PCR

The expression analysis of wild-type and mutated alleles was performed by RT–PCR. Briefly, total RNA was extracted using TRI Reagent (Ambion) accordingly with manufacture’s instruction. cDNA was synthesized starting from 500 ng of RNA.

The following primers were used for RT–PCR: *Pik3c2g* (RTc2gex1-for: 5′-ATTCGATGCTCTACCTCCATC-3′ and RTc2gex3-rev: 5′-TAGCGTGTGGCATAAGAAGG-3′), *lacZ* (forward: 5′-CTGGCGTAATAGCGAAGAGG-3′ and reverse: 5′-TATGCAGCAACGAGACGTC-3′); and *actb* primers (forward: 5′-TGTTACCAACTGGGACGACA-3′ and reverse: 5′-TCTCAGCTGTGGTGGTGAAG-3′) were used as control RT–PCR. For real-time RT–PCR quantification of gluconeogenesis mRNA levels, male mice were fasted overnight and intraperitoneally injected with glucose solution (2 g kg^−1^ of body weight). Total RNA was extracted as above. The following primers were used for Real-Time PCR: *Pck1* (forward: 5′-GAAGAAATGCTTTGCGTTGC-3′ and reverse: 5′-TGCCTTCGGGGTTAGTTATG-3′), *Gck* (forward: 5′-GTGAGGTCGGCATGATTGT-3′ and reverse: 5′-TCCACCAGCTCCACATTCT-3′), *G6pc* (forward: 5′-TCTGTCCCGGATCTACCTTG-3′ and reverse: 5′-GAAAGTTTCAGCCACAGCAA-3′), *Fbp1* (forward: 5′-TATACCCCGCCAACAAGAAA-3′ and reverse: 5′-AAGCTATGGGGTTGCACTCA-3′), *Srebp1c* (forward: 5′-CCAGAGGGTGAGCCTGACAA-3′ and reverse: 5′-AGCCTCTGCAATTTCCAGATCT-3′), *Scd* (forward: 5′-CCAGAATGACGTGTACGAATGG-3′ and reverse: 5′-GCCACACGGCCCAGTTT-3′), *Fas* (forward: 5′-AGAGACGTGTCACTCCTGGACTT-3′ and reverse: 5′-GCTGCGGAAACTTCAGAAAAT-3′), *Pgc1a* (forward: 5′-TGCCTTCATGCTGTGGTAAGTACT-3′ and reverse: 5′-AAAACCCCGCATTTCTAAAGC-3′) *Pgc1b* (forward: 5′-CCTCTCCAGGCAGGTTCAAC-3′ and reverse: 5′-GGCCAGAAGTTCCCTTAGGATAG-3′)

### Histological and biochemical analysis

For body mass and organ weight, 2-month-old male mice were allowed to eat *ad libitum*, were killed and weight of fresh organs was measured.

White adipose tissue was isolated from anaesthetized mice and fixed in 4% paraformaldehyde in PBS, 4 μm paraffin sections were stained by haematoxylin and eosin. Cross-sectional areas of adipocytes were measured using Metamorph software by counting 250 individual cells from random field in the section, per animal. For haematoxylin and eosin and periodic acid-Schiff (PAS) staining, livers were fixed in 4% paraformaldehyde and sectioned in 4 μm paraffin slides. For Oil red O analysis, liver samples from HFD-fed mice were frozen in OCT compound (VWR BDH Prolabo) and 8-μm thick sections were cut using a cryostat. Cut frozen sections were fixed in formalin, stained with Oil red O (Sigma) solution and counterstained with haematoxylin.

For glycogen determination hepatic glycogen was measured as previously described^53^.

### X-gal staining

Fresh tissues were directly frozen in cryostat and sectioned at 8 μm. The sections were fixed with 2% formaldehyde, 0.2% glutaraldehyde, 1 × PBS for 5 min. After fixation, tissue samples were incubated in staining solution (5 mM K_4_Fe(CN)_6_, 5 mM K_3_Fe(CN)_6,_ 2 mM MgCl_2_, 0.01% Triton, 1 mg ml^−1^ X-gal, 0.1 × PBS) at 37 °C, in the dark, overnight. The sections were subsequently washed with PBS and counterstained with nuclear fast red for 5 min.

### Liver extracts preparation

Animals were fasted overnight and insulin (Actrapid, Novo Nordisk) was administrated intraperitoneally (0.75 UI kg^−1^ diluted in PBS solution). Five, 15 or 30 min after injection, tissues were removed, frozen in liquid nitrogen and homogenized in lysis buffer (50 mM Tris-HCl, pH=8, 150 mM NaCl, 1% Triton X-100) supplemented with 50 mM NaF, 2 mM sodium orthovanadate, 1 mM sodium pyro-phosphate and protease inhibitor cocktail (Roche).

### Immunoblotting and immunoprecipitation

Proteins from total liver or cellular lysates or immunoprecipitated were separated by SDS– polyacrylamide gel electrophoresis (SDS–PAGE), and probed with different primary antibodies as specified in each figure legend. The specific signals were amplified by addition of horseradish peroxidase-conjugated secondary antibodies and visualized with enhanced chemiluminescence (ECL from Millipore). Western blotting images were processed using a ChemiDoc XRS digital imaging system with Quantity One 1-D analysis software (Bio-Rad Laboratories, Inc.).

Antibodies from Cell Signaling Technology (working dilution 1:1,000) were the following: phospho-Akt (Ser473) (#4060), phospho-Akt (Ser308) (#13038), phospho-Akt2 (#8599) phospho-GSK3 (Ser9)(#9327), phospho-FoxO1-3 (Thr24/32) (#9464), phospho-p70S6K (Thr389) (#9234), phospho-Glycogen Synthase (Ser641) (#3891), total Akt1 (#2967), total Akt2 (#3063, #5239), total Glycogen Synthase (#3893), total FoxO3 (#12829), total GSK3 (#12456), total p70S6K (#2708), APPL1(#3858), Rab5(#2143) (#3547), Rab7(#2094), Myc-Tag (#2272). Antibody to Glut2 (working dilution 1:1,000) was from Santa Cruz Biotechnology Inc (sc-9,117). Original gel images are shown in Supplementary Fig. 9.

### PtdIns(3,4)*P*_2_ and PtdIns(3,4,5)*P*_3_ localization

Mice were fasted overnight and insulin (Actrapid, Novo Nordisk) was administrated intraperitoneally (0.75 UI kg^−1^ diluted in PBS solution). Fifteen minutes after injection (or 5 min for PtdIns(3,4,5)*P*_3_ detection), liver left lobe was removed and perfused with PBS, followed by 4% paraformaldehyde perfusion. Liver samples were additionally fixed overnight in 4% paraformaldehyde, followed by 25% sucrose overnight at 4 °C. Following fixation, liver samples were frozen in OCT compound (VWR BDH Prolabo) and 8-μm thick sections were cut using a cryostat. Liver sections were equilibrated 5 min in PBS at room temperature and incubated 5 min with 0.02% saponin and then 15 min with 50 mM NH_4_Cl. After 1 h blocking with 10% goat serum at room temperature, sections were incubated with mouse anti-PtdIns(3,4)*P*_2_ antibody (Z-P034b, Echelon Biosciences) at a 1:200 dilution in 0.1% goat serum. For PtdIns(3,4,5)*P*_3_ detection, mouse anti-PtdIns(3,4,5)*P*_3_ antibody (Z-P345, Echelon Biosciences) at a 1:100 dilution was used. After washing with PBS, Alexa 568-conjugated secondary antibody (Alexa Fluor 568 Goat Anti-Mouse IgG, Invitrogen), 1:200 diluted in PBS 0.1% goat serum, was added and incubated 1 h at room temperature. After washing with PBS, the sections were counterstained with 4,6-diamidino-2-phenylindole and mounted.

To test antibody specificity against selected phosphoinositide, liposomes containing both 95% (mol^−1^) phosphatidylserine (PS) and 5% phosphatidylinositol-3,4-bisphosphate diC16 (PtdIns(3,4)*P*_2_diC16; P3416, Echelon Biosciences) or 5% phosphatidylinositol-4,5-bisphosphate diC16 (PtdIns(4,5)*P*_2_diC16; P4516, Echelon Biosciences) were prepared by drying under nitrogen flux the lipid mixtures and resuspending it in 10 mM HEPES, pH 7.5, and EDTA 1 mM, followed by sonication. The anti-PIP(3,4)*P*_2_ antibody (Z-P034b, Echelon Biosciences) was pre-incubated with (PS/PtdIns(3,4)*P*_2_diC16) or (PS/PtdIns(4,5)*P*_2_diC16) liposomes (100 μg) for 1 h at room temperature in PBS at a 1:200 dilution. Following pre-incubation, the antibody was added to the cryosections as above described. To visualize PtdIns(3,4)*P*_2_ in cultured cells, a GFP-TAPP1 probe was used as previously described^54^.

### GS activity assay

GS activity was measured as previously described^55^. Briefly, cleared liver extracts (300 μg of total protein) were incubated with a buffer containing and 0.2 μl of UDP-(^14^C)Glucose (Perkin Elmer, NEC403010UC) in a final volume of 90 μl in the presence or absence of 6.7 mM glucose-6-phosphate. The reaction was incubated for 20 min, spotted on 3M filter paper (Whatman-GE Healthcare), washed and counted.

### EE purification

For subcellular fractionation, liver tissues or cultured cells were gently homogenized in homogenization buffer (250 mM sucrose, 3 mM imidazole pH 7.4, plus protease inhibitor cocktail). The samples were centrifuged at 3,000 r.p.m. to remove nuclei and cell debris. Postnuclear supernatant was subsequently separated by sucrose gradient centrifugation. In detail, the postnuclear supernatants were adjusted to 40.6% sucrose using a stock solution (62% sucrose, 3 mM imidazole pH 7.4), loaded at the bottom of centrifugation tubes (SW55), then sequentially overlaid with 1.5 ml of 35% sucrose solution (35% sucrose, 3 mM imidazole pH 7.4) followed by 1 ml of 25% solution (25% sucrose, 3 mM imidazole pH 7.4) and 1 ml of homogenization buffer on top of the load. After 1 h centrifugation, at 35,000 r.p.m. at 4 °C, EEs were recovered from interphase between 35 and 25% layers, LEs were recovered from uppermost portion of 25% phase, and heavy membranes including endoplasmic reticulum, Golgi and plasma membranes were recovered from lowest interphase. Subsequently proteins from EE and LE fractions were precipitated with methanol/chloroform and loaded in SDS–PAGE for western blot analyses.

### Active Rab5 binding

GST-Rab5A (*Canis lu*p*us*) was expressed in BL21 Star (DE3) bacterial cells (#C6010-03, Life Technologies). Protein was purified with glutathione beads (Thermo Scientific), analysed by SDS–PAGE and Coomassie Blue staining, and used for Rab5A pull-down experiments. PI3K-C2γ and Vps15 were synthesized using the TNT Quick Coupled Transcripiton/Translation Systems (Promega) and Expre^35^s^35^s, [^35^S]-Protein Labeling Mix (Perkin Elmer). GST-Rab5A beads were loaded with either GDP or GTPγS as described^56^, and incubated with the [^35^S]-labelled proteins at 4 ^o^C for 2 h. After six washes with 20 mM Tris (pH 7.4), 25 mM NaCl, 5 mM MgCl_2_, 0.1% NP-40, 1 mM dithiothreitol, 10 μM of either GDP or GTPγS proteins bound to the beads were analysed by SDS–PAGE and autoradiography.

### Immunofluorescence of hepatocytes and cell lines

Hepatocytes were isolated from livers using a modified version of a previously described protocol^57^. Briefly after dissection, liver left lobe was perfused with Hepatocyte Liver Perfusion Medium (Gibco) and collagenase containing medium, Hepatocyte Liver Digest Medium (Gibco). Subsequently to the perfusion, a mechanical disruption of the capsule lobe was performed and the cell suspension was applied over Percoll 1.06 g ml^−1^ solution. The cell layer recovered from the bottom of the falcon tube was washed with Williams' Medium E with GlutaMAX I (Gibco), 4% of Fetal Bovine Serum (Gibco) and 1% Penicillin/Streptomycin (Invitrogen). Cell viability of the enriched hepatocyte preparation was assessed by Trypan blue staining. Freshly isolated hepatocytes were plated onto collagen-coated 12-well glass plates (2 × 10^5^ cells per well), containing Williams' Medium E with GlutaMAX I (Gibco), 4% of Fetal Bovine Serum (Gibco) and 1% Penicillin/Streptomycin (Invitrogen). The following day the medium was changed and hepatocytes were serum-starved overnight in DMEM medium with 1% Penicillin/Streptomycin (Invitrogen) without serum. The next day the hepatocytes were starved with DMEM without glucose, without L-glutamine, without sodium pyruvate, without phenol red (Gennaxon) and, after 4 h, insulin (Actrapid, Novo Nordisk) was added to the medium at 100 nM concentration.

For electroporation, the Amaxa Nucleofector was used with the mix specifically optimized for primary hepatocyte transfection (Amaxa #VPL-1,004) following instructions from the manufacturer. Cells were analysed by immunofluorescence 24 h after transfection. Immunofluorescence was performed with the antibodies mentioned above but at a working dilution of 1:100.

### Glucose tolerance test

GTT was conducted on 2-month-old male mice or 8-month-old mice, fasted overnight, by intraperitoneal injection of glucose dissolved in PBS solution (2 mg kg^−1^ of body weight). Blood glucose concentration was determined using glucometer and Accu-Chek Active strips (Roche).

### Insulin tolerance test

ITT was conducted on 2-month-old male mice fasted for 6 h before intraperitoneal injection of 0.75 UI kg^−1^ of human insulin (Actrapid, Novo Nordisk) diluted in saline solution. Blood glucose was measured as for GTT.

### Pyruvate tolerance test

Pyruvate tolerance test was conducted on 2-month-old male mice fasted for 6 h before intraperitoneal injection of 2 g kg^−1^ pyruvate diluted in saline solution. Blood glucose was measured as for GTT.

### Food consumption and activity cage

For food intake determination, 2-month-old male mice were placed individually in cages with free access to food and water and the food consumption was measured over 1 week. For physical activity, horizontal and vertical movements of mice individually placed in activity cage (Ugo Basile Instruments) were measured over a period of 24 h.

### MRI and metabolic analysis on HFD-fed mice

For metabolic analysis on HFD-fed animals, male mice at 1 month of age were fed *ad libitum* with fat-rich diet (60% energy from fats, ETPF4215R0M Mucedola). After 4 months of treatment, circulating levels of glucose and insulin and GTT analysis were performed as reported above.

For determination of body composition by MRI, images were acquired on a 1 Tesla M2 system (Aspect, Israel) equipped with a 30-mm transmitter/receiver (TX/RX) solenoid coil and NRG Console 2.0 software. Mice’s breathing was monitored with a circular pneumatic pillow under the abdomen (SA Instruments). A T_1-weighted_ Spin-Echo sequence was used to acquire high-resolution whole-body coronal images (repetition time/echo time/flip angle/number excitations [TR/TE/FA/NEX]=400 ms/8.7 ms/90°/3; field of view [FOV]=10 cm, matrix=192 × 192, number of slices: 18, slice thickness: 1.5 mm, in-plane spatial resolution: 521 μm, acquisition time: 4 min).

All data processing was performed by in-house script implemented in a commercial software package (MATLAB R2008, The MathWorks Inc.). The T_1-weighted_ image histogram has three dominating classes, background, lean mass and fat, so the total fat volume was isolated by segmenting the image into three categories by using a k-means clustering algorithm.

### Plasma glucose and insulin levels

For determination of blood levels of glucose and insulin, male mice fed *ad libitum* were analysed. For glucose determination in fed conditions, measurements in triplicate at the same hour of three different days were performed with each animal. For fasted levels of glucose and insulin, mice were starved overnight. Insulin levels were determined using RIA kit, following the manufacturer’s instructions (Sensitive Rat insulin RIA, Millipore).

### Statistical analysis

Statistical significance was calculated with Student’s *t*-test and one- or two-way analysis of variance followed by Bonferroni’s multiple comparison post-test. Values are reported as the mean±standard error of the mean. Statistical significance is indicated as: *P<0.05; **P<0.01; ***P<0.001.

## Additional information

**How to cite this article:** Braccini, L. *et al.* PI3K-C2γ is a Rab5 effector selectively controlling endosomal Akt2 activation downstream of insulin signalling. *Nat. Commun.* 6:7400 doi: 10.1038/ncomms8400 (2015).

## Supplementary Material

Supplementary Information Supplementary Figures 1-9
