# Supplementary material for: PI3K-C2γ is a Rab5 effector selectively controlling endosomal Akt2 activation downstream of insulin signalling
Source: Nat Commun. 2015 Jun 23;6:7400. doi: 10.1038/ncomms8400 (PMC4479417; doi:10.1038/ncomms8400)
Supplement: Supplementary Information — Supplementary Figures 1-9 [file ncomms8400-s1.pdf]

Supplementary Fig. 1

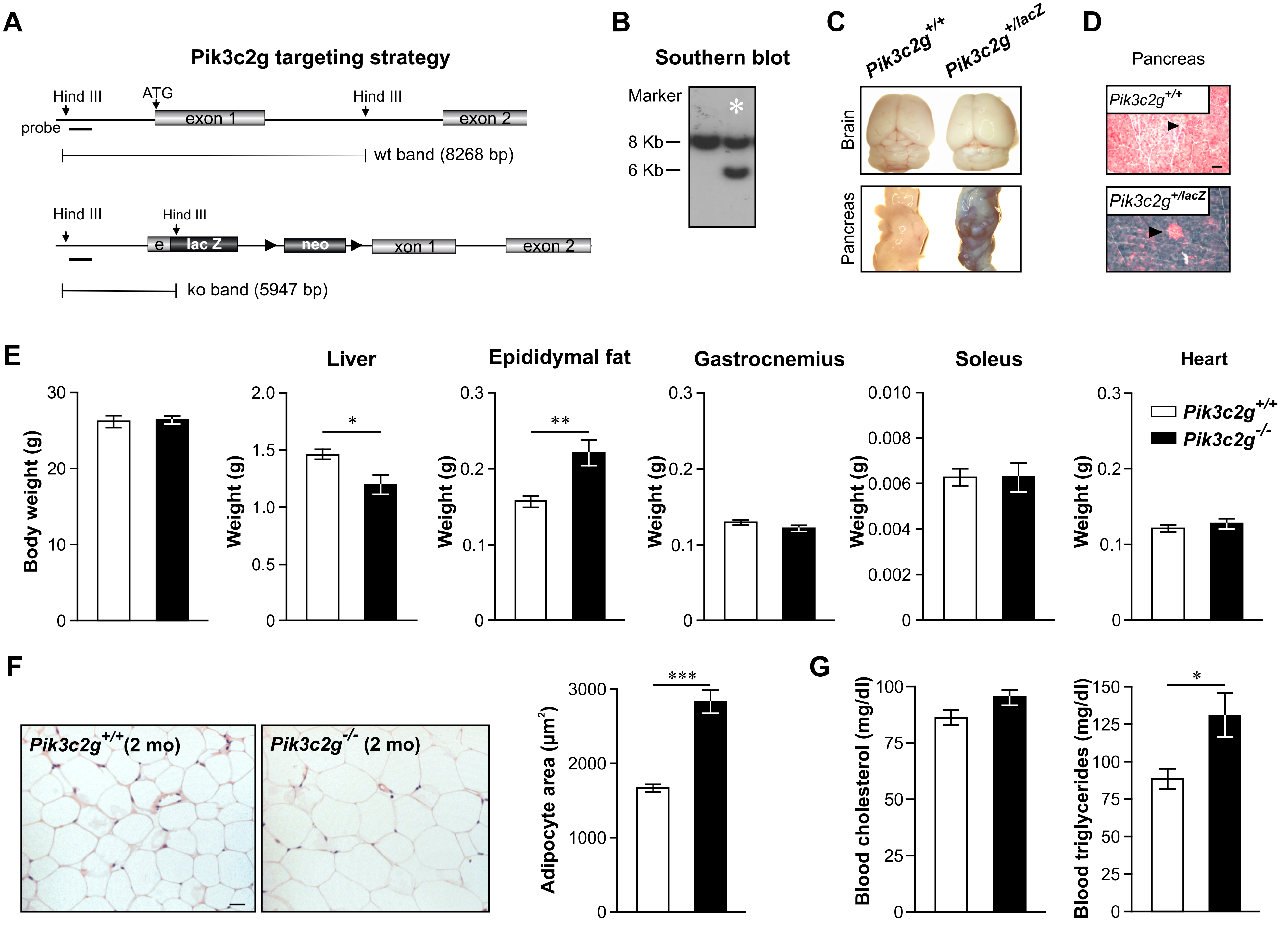

Supplementary Figure 1. Gene targeting of the *Pik3c2g* locus and its effect on selected insulin-sensitive organs

(a) Schematic description of gene-targeting strategy for PI3K-C2γ ablation. The cassette including *lacZ* coding sequence and neomycin resistance (neo) is shown (not in scale).

(b) Southern blot validation of targeted clones. Endonuclease *HindIII* restriction sites for Southern blot analysis on ES are also reported. The autoradiogram represents 1 (*) out of 10 positive clones.

(c) Whole-mount LacZ staining of organs from heterozygous (*Pik3c2g*$^{+/-}$) mice and controls (*Pik3c2g*$^{+/+}$) imaged *in toto*.

(d) Representative LacZ staining of pancreas sections from *Pik3c2g*$^{+/+}$ and heterozygous *Pik3c2g*$^{+/-}$ mice. Insulin producing islets of Langerhans (arrowhead) are not stained in *Pik3c2g*$^{+/-}$ sections. Scale bar 100 μm.

(e) Whole-body and selected insulin-sensitive organ weight of *Pik3c2g*$^{+/+}$ and *Pik3c2g*$^{-/-}$ mice (n=8 and n=5, respectively).

(f) Representative histological sections of epididymal fat pads stained with hematoxylin-eosin. Scale bar 40 μm. Cross-sectional area of adipocytes from 2-month-old *Pik3c2g*$^{+/+}$ and *Pik3c2g*$^{-/-}$ mice is reported in the bar graph on the right.

(g) Blood lipid levels in 2-month-old *Pik3c2g*$^{+/+}$ and *Pik3c2g*$^{-/-}$ mice measured in fasted condition (n=11 and n=13 respectively).

Results represent mean±s.e.m. *p<0.05, **p<0.01 mutant vs. the respective wild-type controls. p values were determined using Student's t test.

Supplementary Fig. 2

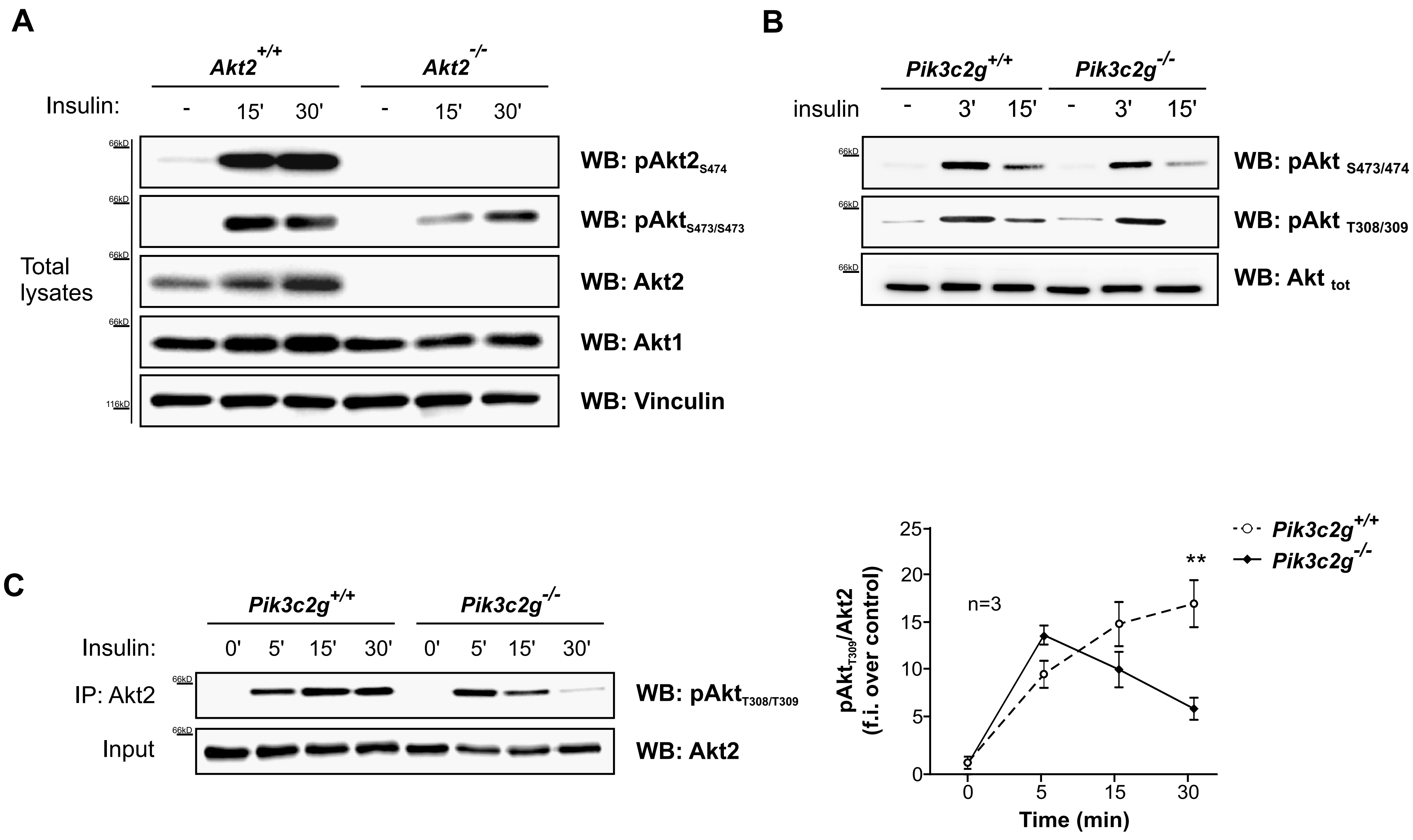

Supplementary Figure 2. Loss of PI3K-C2γ impairs hepatic Akt2 phosphorylation on both Thr308 and Ser473

(a) Akt 1 and Akt2 antibody specificity assessment. Liver extracts from Akt2-deficient ($Akt2^{-/-}$) mice and wild-type controls ($Akt2^{+/+}$) before and after insulin stimulation for the given times were analyzed with the listed antibodies. Note that, differently from the pan-pAkt antibody recognizing Ser473 on Akt1 as well as Ser 474 on Akt2, the antibody against pAkt2 Ser474 does not provide a signal in $Akt2^{-/-}$ samples.

(b) Western blot analysis of Akt phosphorylation upon 100nM insulin stimulation of primary hepatocytes purified from 2-month-old *Pik3c2g*$^{+/+}$ and *Pik3c2g*$^{-/-}$ mice. Immunoblot is representative of 4 independent experiments.

(c) Analysis of Akt2 phosphorylation on Thr309 after immunoprecipitation with antibodies selectively recognizing Akt2. An example of 3 independent experiments providing equal results is shown on the left. Quantification of Akt2 phosphorylation in *Pik3c2g*$^{+/+}$ and *Pik3c2g*$^{-/-}$ livers is shown on the right.

Results represent mean±s.e.m of the number of replicates (n) reported in the figure. **$p<0.01$ mutant vs. the respective wild-type controls. p values were determined using 2 way ANOVA followed by Bonferroni post-hoc test.

Supplementary Fig. 3

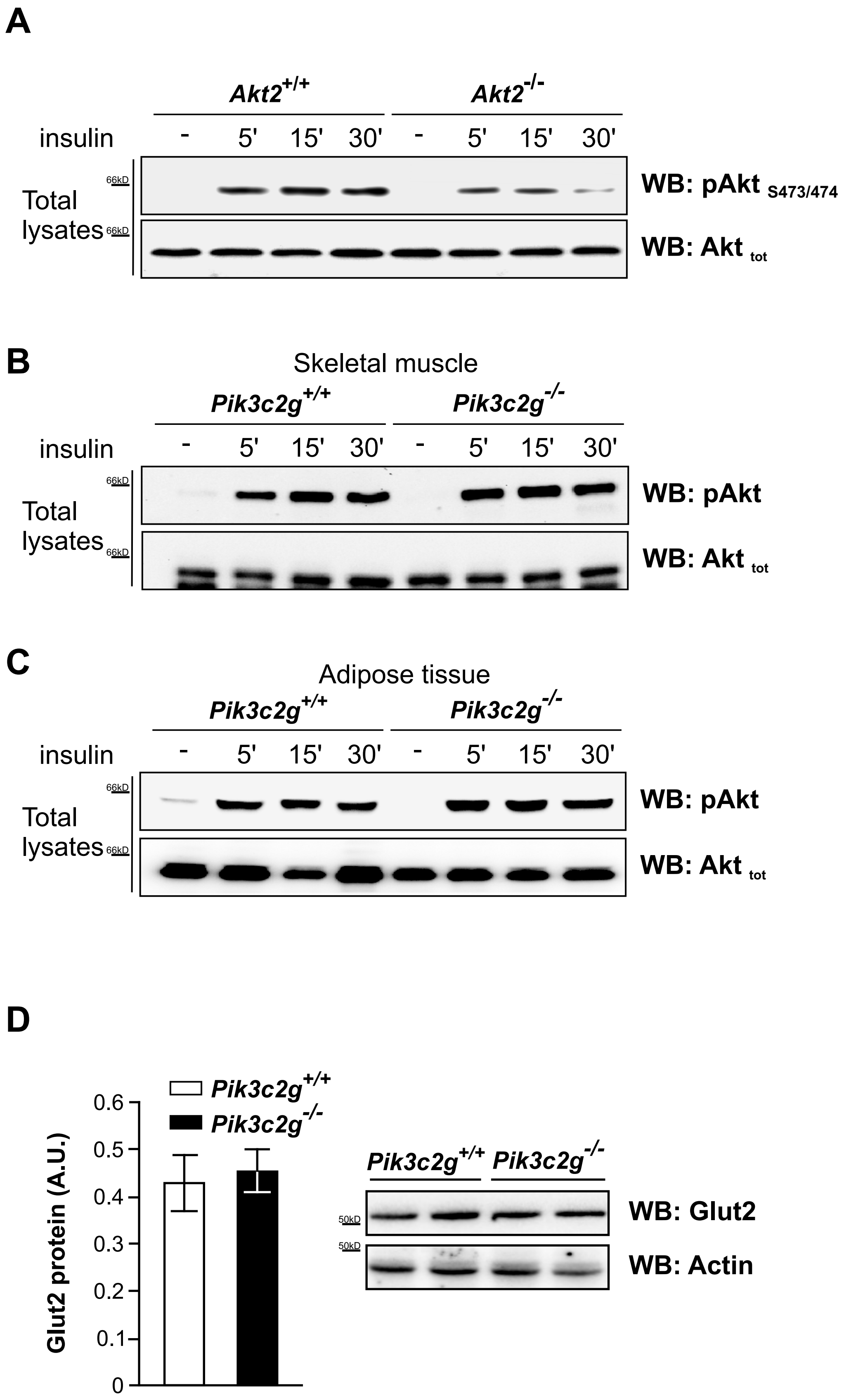

Supplementary Figure 3. Loss of PI3K-C2γ impairs hepatic Akt2 phosphorylation but not gluconeogenesis

(a) Representative Western blot of 3 replicates showing of Akt phosphorylation on Ser473 in primary Akt2$^{+/+}$ and Akt2$^{-/-}$ hepatocytes stimulated with insulin as indicated. Note that the loss of Akt2 reduces total pSer473-Akt only at 30min, compatibly with the loss of PI3K-C2γ specifically affecting prolonged and sustained Akt2 phosphorylation shown in Figure 2.

(b, c) The insulin-dependent prolonged and sustained Akt phosphorylation on Ser473 is preserved in skeletal muscles (b) and adipose tissue (c) of *Pik3c2g*$^{-/-}$ mice. Shown are representative blots of two independent experiments for each of the two tissues.

(d) Quantification of Glut2 expression in *Pik3c2g*$^{+/+}$ and *Pik3c2g*$^{-/-}$ livers. Results represent mean±s.e.m. Statistical significance was determined using Student's t test and no statistically significant difference was found between wild-type and mutant samples. The right panel show a representative blot out of 3 blots with 2 individuals of each genotype.

Supplementary Fig. 4

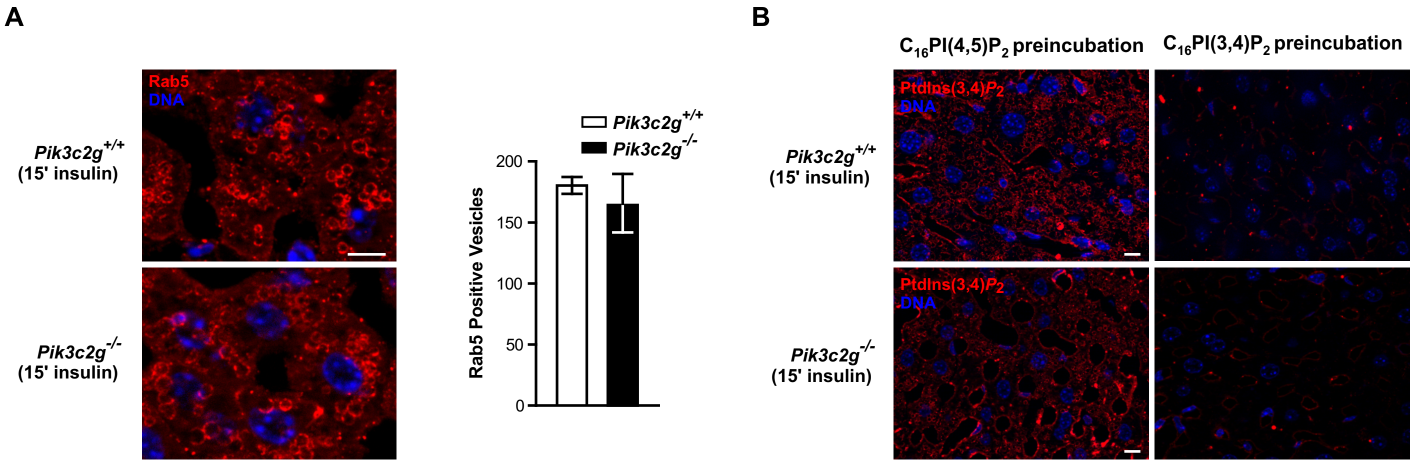

Supplementary Figure 4. PI3K-C2γ activity in early endosomes

(a) Rab5 staining of liver sections of insulin stimulated *Pik3c2g*$^{+/+}$ and *Pik3c2g*$^{-/-}$ mice. Scale bar equals to 10µm. Quantification of five high magnification fields per mouse (n=3) is represented in the right panel. Results represent mean±s.e.m. Lack of statistical difference was determined using Student's t test.

(b) The anti-PtdIns(3,4)P_2 antibody was pre-incubated with liposomes containing 5% phosphatidylinositol-4,5-bisphosphate diC16 or with 5% phosphatidylinositol-3,4-bisphosphate diC16. After 1 hour of incubation with liposomes, the antibody was used for immunofluorescence assay on liver section from *Pik3c2g*$^{+/+}$ and *Pik3c2g*$^{-/-}$ insulin treated mice as described in Figure 4. Scale bar equals to 10µm.

Supplementary Fig. 5

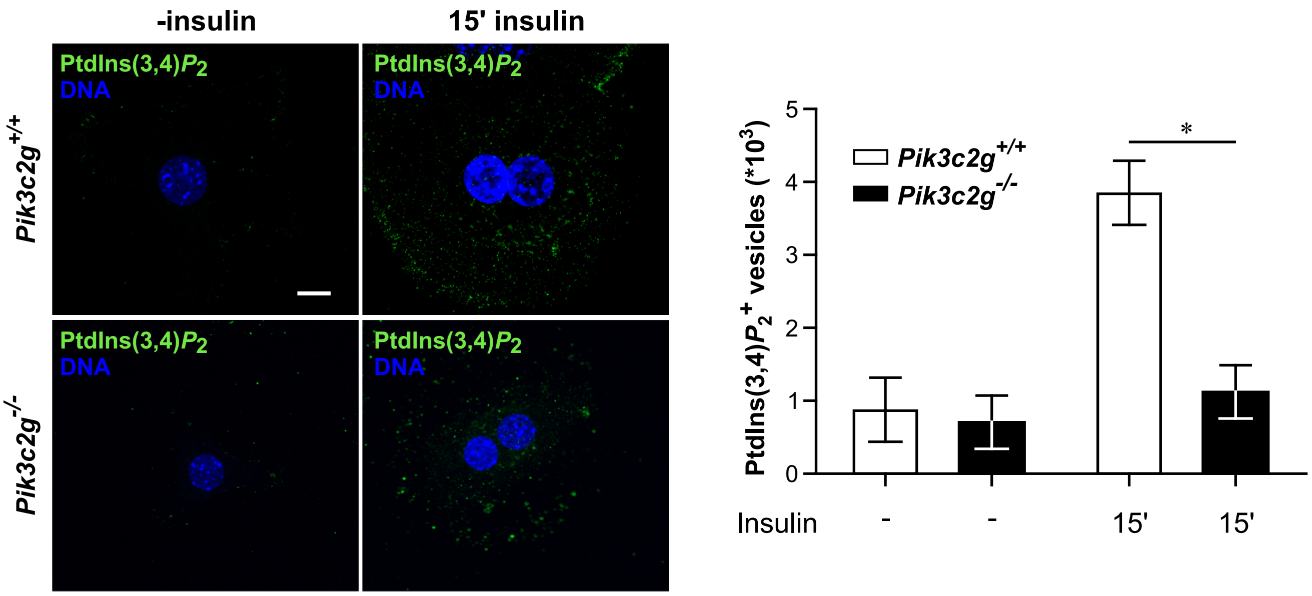

Supplementary Figure 5. Loss of PI3K-C2γ reduces PI(3,4)P2

Pik3c2g$^{+/+}$ and *Pik3c2g*$^{-/-}$ primary hepatocytes were stained with anti-PtdIns(3,4)P_2 antibodies and photographed before and after 15min stimulation with insulin. PtdIns(3,4)P_2 -positive vesicles were counted in 10 cells per genotype (right panel). Scale bar equals to 10µm.

Results represent mean±s.e.m. *$p<0.05$ mutant vs. the respective wild-type controls. p values were determined using one way ANOVA followed by Bonferroni post-hoc test.

Supplementary Fig. 6

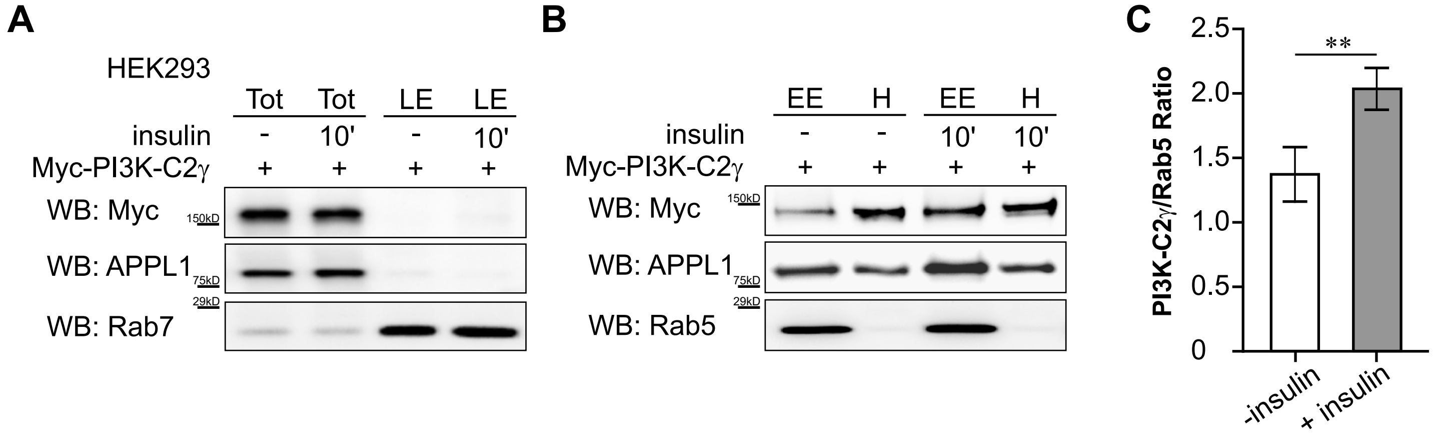

Supplementary Figure 6. Rab5-dependent PI3K-C2γ cellular distribution

(a) Distribution of PI3K-C2γ in subcellular fractions before and after insulin stimulation. PI3K-C2γ does not localize in Rab7-positive late endosome (LE). The immunoblots are representative of 4 independent experiments.

(b) Distribution of PI3K-C2γ in early endosome (EE) and in cytosolic/heavy membranes (h) fractions from the same experiment shown in a. Note that PI3K-C2γ is enriched in EE after insulin stimulation. The immunoblots are representative of 4 independent experiments.

(c) Quantification of PI3K-C2γ on Rab-5 positive EE.

Results represent mean±s.e.m. **$p<0.01$ starved vs. insulin-stimulated cells. p values were determined using Student's t test.

Supplementary Fig. 7

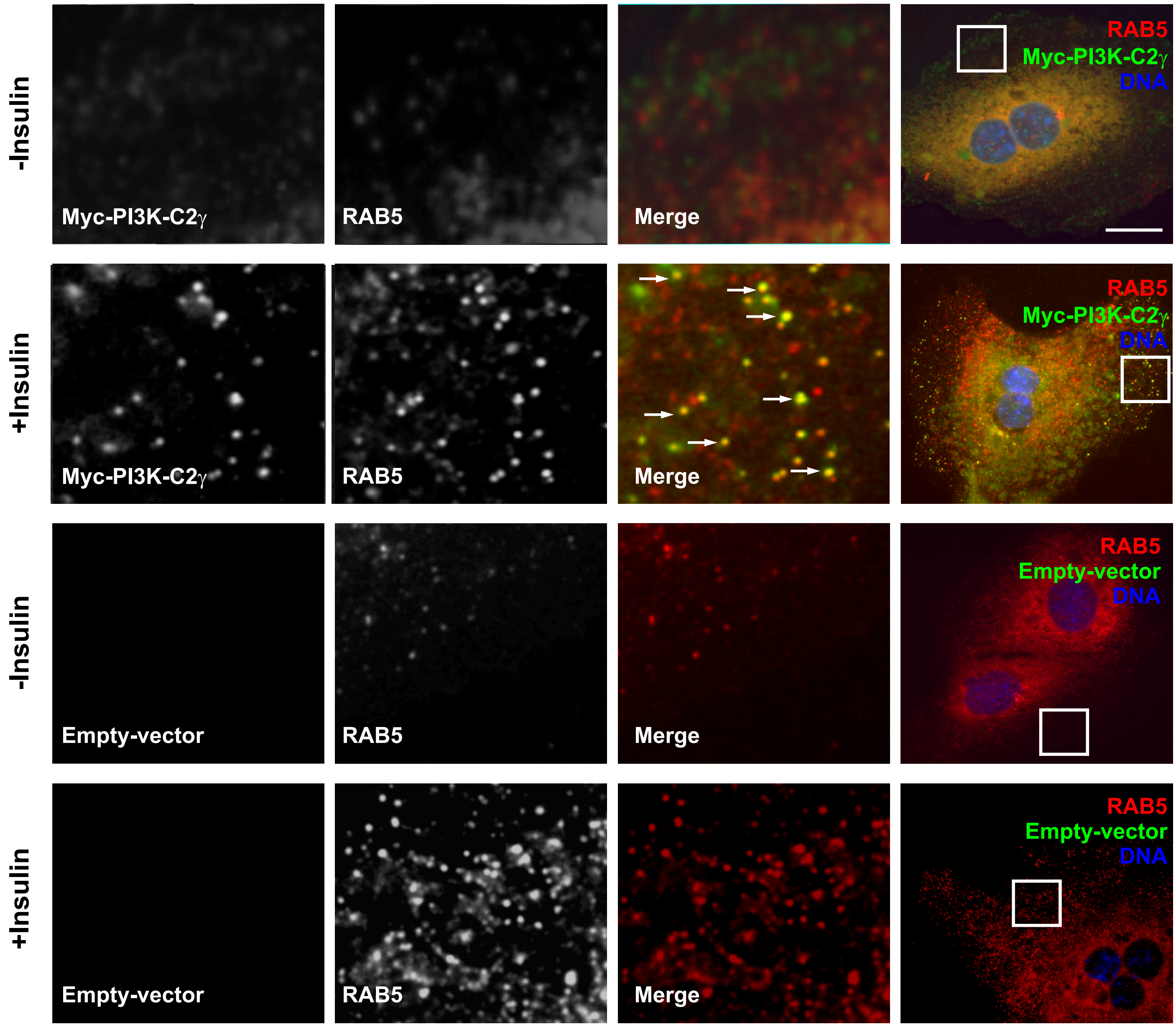

Supplementary Figure 7. PI3K-C2γ and Rab5 co-localize in insulin stimulated hepatocytes

Murine primary hepatocytes were transfected with the Amaxa Nucleofector to express either the Myc-tagged PI3K-C2γ (upper panels) or the empty vector (lower panels). Cells were stained after overnight starvation (-insulin) and after 15 min of insulin stimulation (+insulin) with Myc (green) and Rab5 (red) antibodies. Note that no staining is evident with Myc antibodies when cells are transfected with the empty vector. Bars equal to 10 µm and 1 µm (inset).

Supplementary Fig. 8

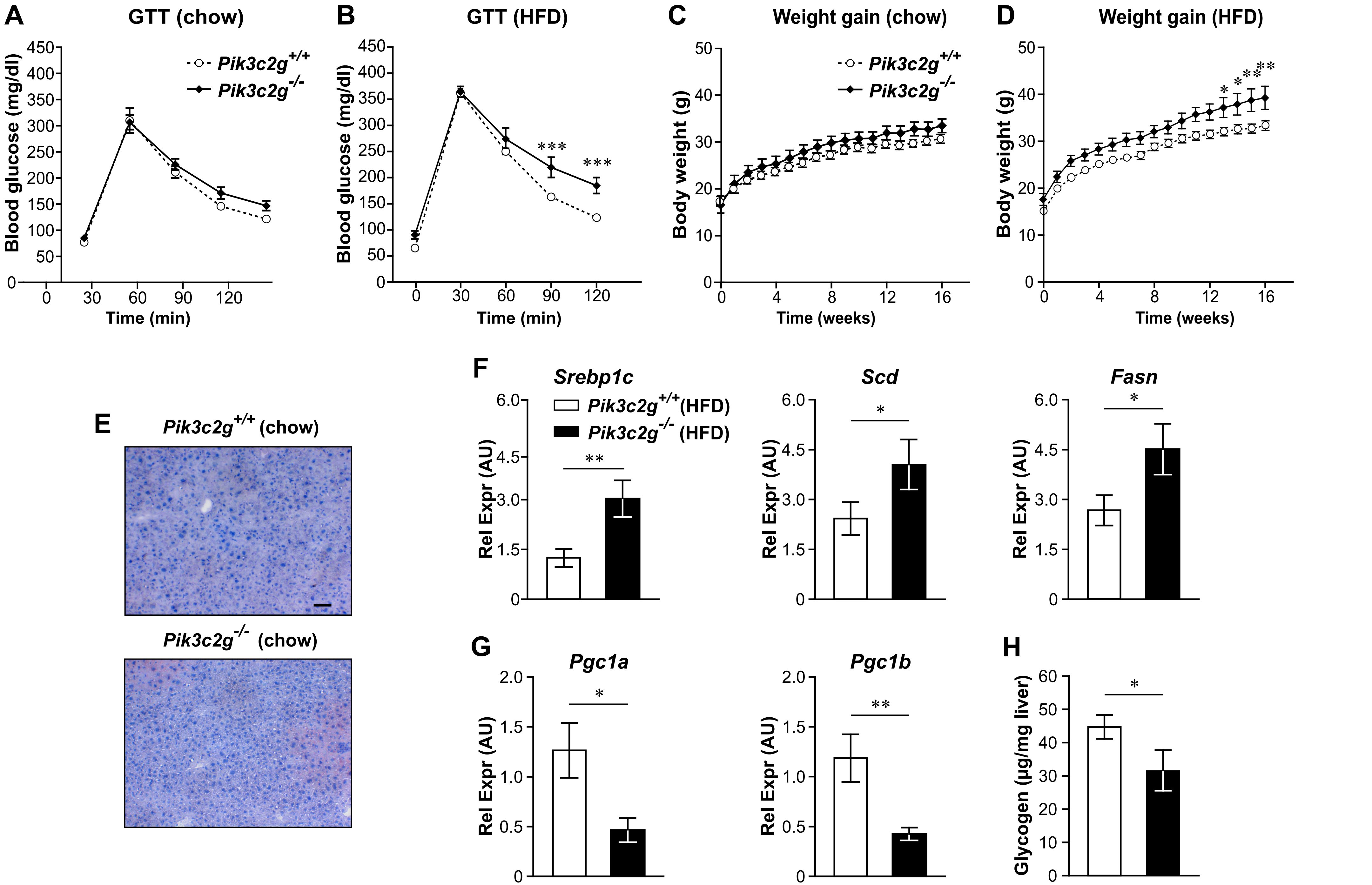

Supplementary Figure 8. The lipogenic pathway is altered in HFD-fed *Pik3c2g*$^{-/-}$ mice

(a, b) Glucose tolerance test (GTT) in normal chow diet (a) and high fat diet (HFD) (b) -fed *Pik3c2g*$^{+/+}$ (open circles) and *Pik3c2g*$^{-/-}$ (filled diamonds) mice (n=11 and n=9, respectively)
(c, d) Analysis of body weight, monitored weekly in *Pik3c2g*$^{+/+}$ and *Pik3c2g*$^{-/-}$ mice fed with normal chow diet (c) or high fat diet (d) for 16 weeks (n=14 and n=9, respectively).
(e) Livers of mice fed on normal chow diet do not develop fatty liver. Representative Oil red O-stained liver sections from chow diet-fed *Pik3c2g*$^{+/+}$ and *Pik3c2g*$^{-/-}$ mice are shown. Scale bar 100μm.
(f, g) Hepatic expression of genes involved in lipid biosynthesis (f) and lipid catabolism (g), determined by real-time PCR in livers from *Pik3c2g*$^{+/+}$ and *Pik3c2g*$^{-/-}$ mice fed with HFD for 16 weeks (n=11 and n=9, respectively).
(h) Measurement of glycogen in livers from *Pik3c2g*$^{+/+}$ and *Pik3c2g*$^{-/-}$ mice fed with HFD for 16 weeks (n=4 and n=4, respectively).
Results represent mean±s.e.m. *p<0.05, **p<0.01, *p<0.001 mutant vs. the respective wild-type controls. p values were determined using 2 way ANOVA followed by Bonferroni post-hoc test (a-d) and Student’s t test (f-h).

Supplementary Fig. 9

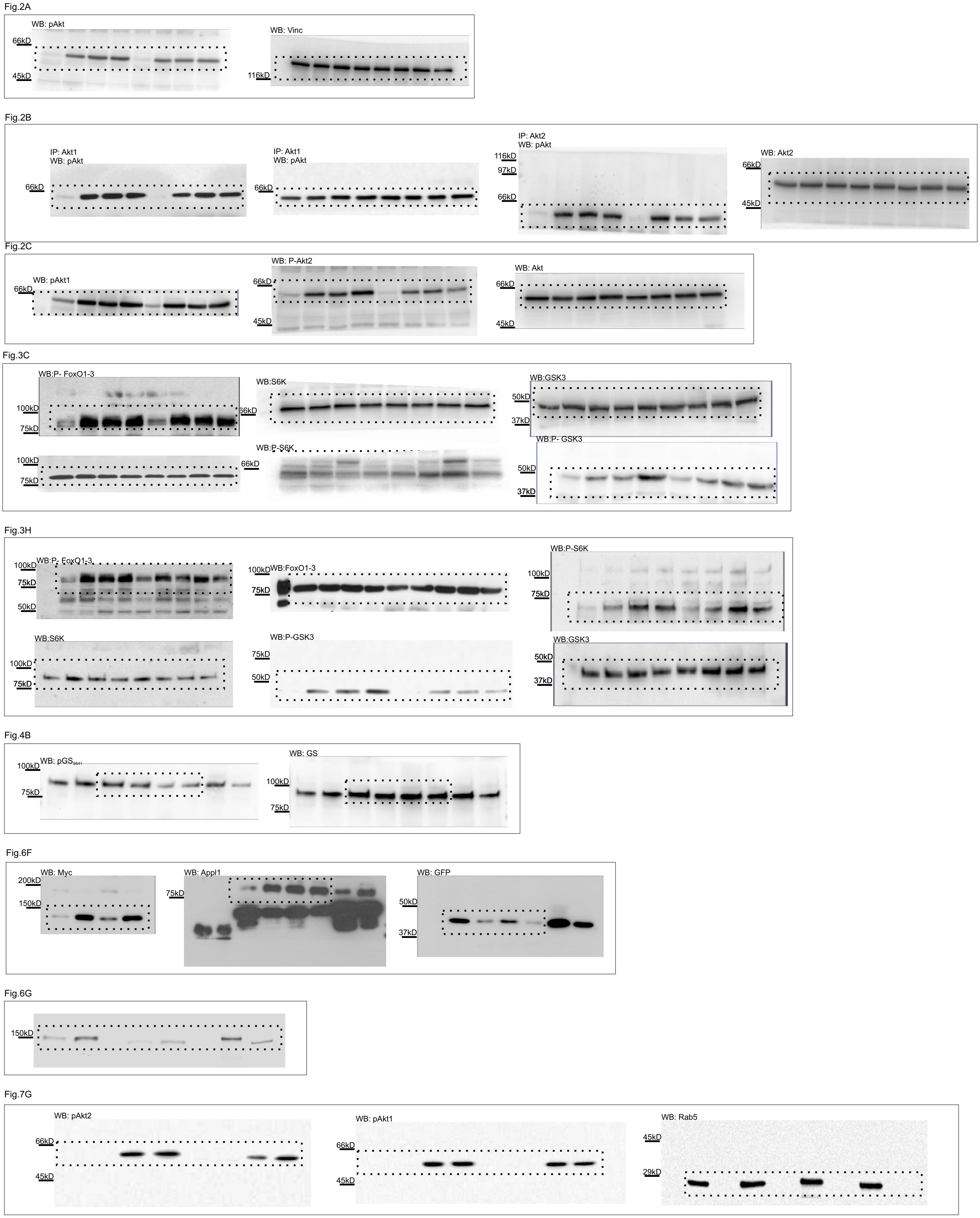

Supplementary Figure 9.

Lists of original gel images of western blot analysis. Boxes highlight lanes used in figures.
